# Varied microbial community assembly and specialization patterns driven by early life microbiome perturbation and modulation in young ruminants

**DOI:** 10.1093/ismeco/ycae044

**Published:** 2024-04-09

**Authors:** Zhe Pan, Tao Ma, Michael Steele, Le Luo Guan

**Affiliations:** Department of Agricultural, Food and Nutritional Science, University of Alberta, Edmonton, AB T6G 2P5, Canada; Key Laboratory of Feed Biotechnology of the Ministry of Agriculture and Rural Affairs, Institute of Feed Research, Chinese Academy of Agricultural Sciences, Beijing 100081, China; Department of Animal Biosciences, University of Guelph, Guelph, ON N1G 2W1, Canada; Department of Agricultural, Food and Nutritional Science, University of Alberta, Edmonton, AB T6G 2P5, Canada; Faculty of Land and Food Systems, The University of British Columbia, Vancouver, BC V6T 1Z4, Canada

**Keywords:** determinism, stochasticity, specialization, community assembly, calf, diarrhea, probiotics

## Abstract

Perturbations and modulations during early life are vital to affect gut microbiome assembly and establishment. In this study, we assessed how microbial communities shifted during calf diarrhea and with probiotic yeast supplementation (*Saccharomyces cerevisiae var. boulardii*, SCB) and determined the key bacterial taxa contributing to the microbial assembly shifts using a total of 393 fecal samples collected from 84 preweaned calves during an 8-week trial. Our results revealed that the microbial assembly patterns differed between healthy and diarrheic calves at 6- and 8-week of the trial, with healthy calves being stochastic-driven and diarrheic calves being deterministic-driven. The two-state Markov model revealed that SCB supplementation had a higher possibility to shift microbial assembly from deterministic- to stochastic-driven in diarrheic calves. Furthermore, a total of 23 and 21 genera were specific ecotypes to assembly patterns in SCB-responsive (SCB-fed calves did not exhibit diarrhea) and nonresponsive (SCB-fed calves occurred diarrhea) calves, respectively. Among these ecotypes, the area under a receiver operating characteristic curve revealed that *Blautia* and *Ruminococcaceae* UCG 014, two unidentified genera from the *Ruminococcaceae* family, had the highest predictiveness for microbial assembly patterns in SCB-responsive calves, while *Prevotellaceae*, *Blautia*, and *Escherichia-Shigella* were the most predictive bacterial taxa for microbial assembly patterns in SCB-nonresponsive calves. Our study suggests that microbiome perturbations and probiotic yeast supplementation serving as deterministic factors influenced assembly patterns during early life with critical genera being predictive for assembly patterns, which sheds light on mechanisms of microbial community establishment in the gut of neonatal calves during early life.

## Introduction

Emerging evidence has highlighted that the establishment of the gut microbiome is critical in neonatal mammals because it contributes to the development of the immune system and maintenance of the health and function of the host [1–3]. Establishing a stable and healthy gut microbiome not only affects nutrient absorption and host metabolism but also has a life-long impact on host health [4, 5]. Therefore, researchers have proposed that early life is the best window to manipulate the gut microbiome to achieve a long-term effect on host health [1]. To date, although many findings have revealed the dynamics and shifts of the gut microbiome during this stage, the key ecological drivers regulating microbiome assembly (or formation) have not been elucidated to gain a comprehensive understanding of the microbial establishment process in neonatal mammals.

The microbial establishment process is critical, as early-arriving microbes could hinder or facilitate the colonization of late-arriving microbes (termed the priority effect) [6, 7]. Such a process can directly influence gut microbiome assembly and long-term gut microbiota stability and resilience [8]. The microbial colonization process in the mammalian gut has been proposed to follow the neutral theory that microbial community assembly is driven by both deterministic and stochastic processes [8–12]. In particular, stochastic processes refer to ecological processes that can generate comparable community patterns compared with those generated by random chance alone [13]. This theory has been proven to explain species abundance dynamics; however, the in-depth assessment to adopt this theory to understand the microbiome in the mammalian gut is limited. A recent study revealed the stochastic process in the rumen of adult dairy cows, and stochasticity was acted locally in affecting rumen microbiome development in cattle, which was associated with the different initial microbiome in the first 24 h of life following the two modes of delivery (C-section and natural birth) [14]. Further assessments in this study revealed that both age and diet serve as deterministic factors that act globally affecting rumen microbiome development independent of the mode of delivery [14]. For instance, compositional changes in the rumen microbiome were directly linked to the animal’s age, of which researchers can distinguish the effects of animal development (age) from the effects of diet [14]. This study suggests that neutral theory-based microbiome assembly exists in the rumen, however, whether the neutral theory can affect microbiome assembly and which factors influence microbial assembly in the gut of young ruminants have not been studied.

During early life, the microbial community structures and functions in calves are susceptible to perturbations such as diarrhea that can cause low microbial richness and decrease the functional capacities in carbohydrate and amino acid metabolism [6, 7], leading to decreased growth rate [15]. Recent studies have suggested that probiotic administration could alleviate diarrhea and restore gut microbial diversities, i.e. the supplementation of *Limosilactobacillus reuteri* (previously named as *Lactobacillus reuteri*) could alleviate *Escherichia coli*-induced diarrhea and restore the relative abundance of beneficial microbes including *Lactobacillus*, *Bifidobacterium*, etc. in mice [16]. Feeding S*accharomyces boulardii* mafic 1701 could alleviate intestinal inflammation evidenced by the decreased levels of pro-inflammatory cytokines interleukin-6 and modulate gut microbiota by increasing cecal concentration of microbial metabolites (isobutyrates and valerate) in weaned piglets [17]. Similarly, previous studies also observed that probiotic supplementation could alleviate microbial perturbations caused by diarrhea [6, 18, 19], i.e. supplementing *Saccharomyces cerevisiae var. boulardii* (SCB) decreased the severity of diarrhea from 28.6% to 9.5% and reduced the relative abundance of *Escherichia-Shigella* (an etiological agent causing calf diarrhea) in the gut of neonatal calves [20].

Although microbial profiles from calves with different health conditions or diets are inherently diverse [7, 21], the mechanisms and factors regulating different microbial profiles remain undetermined. We hypothesized that both stochastic and deterministic processes participate in microbiome assembly in the gut of dairy calves during early life and that these processes are affected by calf diarrhea or the use of probiotics (e.g. SCB) with different changing patterns. Furthermore, we speculated that microbial specialization patterns associated with microbial assembly are inherently different due to the gut microbiome modulations. Besides, we assessed how host health accompanied by microbial community structure variations and microbial interactions affect assembly patterns using a subgroup of healthy and diarrheic calves. Moreover, assembly patterns in response to probiotic yeast supplementation under different host health conditions were investigated. Lastly, microbial specialization patterns and bacterial taxa that were predictive for microbial assembly patterns were identified. To achieve these goals, the microbial assembly patterns were evaluated using 393 fecal samples collected from 84 preweaned calves during the first 8 weeks of life.

## Materials and methods

### Animal trial and sample collection

The animal trial was conducted following the Canadian Council of Animal Care and was approved by the Animal Care and Use Committee, the University of Alberta with Animal Care Committee (protocol number AUP00001595). The detailed animal study and sampling information were described by Ma *et al.* [22]. Briefly, 84 Holstein male veal calves aged 6 ± 3 d (±SD) were transported to the veal facility on the same day. Their health conditions and fecal scores were assessed to ensure that each individual did not have signs of disease, injury, and dehydration and recorded over the 8-week period (starting from week 1 to week 8, Fig. S1, see online supplementary material for a colour version of this figure). All calves were only fed milk replacer contained 260 g/kg of crude protein, 160 g/kg of crude fat, and 4.58 Mcal/kg of metabolizable energy on a dry matter basis (Grober Animal Nutrition, Cambridge, Ontario, Canada) during this trial. A total of 42 calves were randomly assigned to the treatment group feeding milk replacer with five grams of live SCB in every afternoon feeding [22]. The other 42 fed milk replacer with a consistent 5 g/day of an inert material of powdered silica (Perkasil SM 660, Grace, Dueren, Germany) as the placebo (CON).

The calves were grouped as diarrheic and healthy based on the fecal scores across the 8-weeks trial. The fecal score was given within 30 min of excretion and the standard health scoring system was used to assess diarrhea according to the following scale: 0 = normal; 1 = semi-formed, pasty; 2 = loose but stays on top of mat; and 3 = watery, sifts through the mat [23]. The trimethoprim sulfa (Trimidox, 0.7 ml/10 kg BW, Vetoquinol) was given for 3 consecutive days when severe diarrhea was detected [22]. Calves identified as diarrhea (watery stool for at least 2 days, fecal score ≥ 2) were assigned as the “unhealthy” group (UH, *n* = 49) and were treated with oral electrolytes (Calf Lyte II, Vetoquinol, Lavaltrie, QC, Canada) with a 3-day trimethoprim sulfa (Trimidox, 0.7 ml/10 kg BW, Vetoquinol) treatment. While calves who did not exhibit diarrhea during the experimental period were designated as the “healthy” group (H, *n* = 35). Particularly, calves in the CON group were divided into two subgroups: healthy (CON-H, *n* = 14) and unhealthy (CON-UH, *n* = 28). Similarly, within the SCB group, 21 calves were assigned to the healthy group (SCB-H, *n* = 21), and the unhealthy group (SCB-UH, *n* = 21, Supplementary file 1). A total of 393 fecal samples (~20 g/sample, *n*_w1_ = 81, *n*_w2_ = 81, *n*_w3_ = 80, *n*_w6_ = 76, *n*_w8_ = 75) were collected from the rectum of calves at weeks 1, 2, 3, 6, and 8 (21 samples were not collected due to the death of two calves from SCB-UH and six calves from CON-UH and six samples due to limited rectum contents collected) and were used for DNA extraction and microbial profiling.

### Amplicon sequencing and microbial community analysis

Total DNA was extracted from 0.2 ± 0.1 g powdered fecal samples using repeated bead beating and a column (RBBC) method [24] and purified using the QIAmp Stool Mini Kit (Qiagen, Hilden, Germany). To generate bacterial compositional profiles, the bacterial V1-V3 region of the 16S rRNA gene was amplified using the bacterial primers Ba9F (5′-GAGTTTGATCMTGGCTCAG-3′) and Ba515Rmod1 (5′-CCGCGGCKGCTGGCAC-3′) [25]. The polymerase chain reaction amplification products were verified using agarose gel (1%) electrophoresis and purified with a Qiagen Gel Extraction Kit (Qiagen, Germany). Negative controls without sample DNA were included in amplicon sequencing. All amplicon libraries were sequenced using an Illumina MiSeq PE 300 platform (2 × 300 pair-end) at Génome Québec, McGill University (Quebec, Canada).

The raw sequence data were processed using QIIME2 (Version 2019.10) [26]. Quality control, denoising, removal of chimeric sequences, and generation of amplicon sequencing variants (ASVs) were performed using the QIIME2 plugin DADA2 [27]. The “decontam” package in R was used to identify contaminating taxa with a higher prevalence in control samples than in true samples [28]. Three ASVs were identified from negative control samples (two *E. coli* taxa and one uncultured bacterium) and were removed from sequence data for all samples. Taxonomic classification was performed in QIIME2 using a taxonomic classifier with the SILVA database (version 132) as the reference.

### Assessment of microbial community assembly patterns

The standardized effect size (SES) index [29] was used to interpret general community assembly patterns, which can be calculated below


\begin{equation*} \mathrm{SES}=\frac{{\mathrm{Cscore}}_{\mathrm{observed}}-{\mathrm{Cscore}}_{\mathrm{null}}}{\mathrm{SD}}[30]. \end{equation*}

Here, ${\mathrm{Cscore}}_{\mathrm{observed}}$ or ${\mathrm{Cscore}}_{\mathrm{null}}$ refers to the checkboard score, which quantifies the genus cooccurrence from the empirical binary matrix (presence/absence) or randomized matrix (null model), respectively. The $\mathrm{Cscore}$ was computed based on 10 000 simulations and sequential swap randomization using the “EcoSimR” package in R. The $\mathrm{SD}$ refers to the standard deviation of ${\mathrm{Cscore}}_{\mathrm{null}}$ [30]. The higher (>2) or lower SES (<−2) value than the expected null value is considered as overdispersion or under dispersion, respectively [30]. The magnitude of SES is considered the strength of the deterministic effect on the community assembly [30].

Besides, the modified Raup–Crick distance (${\beta}_{\mathrm{RC}}$), which was constructed from a probabilistic null model, was used to assess community assembly patterns [13]. The null model simulation is constructed to represent the pure stochastic-driven community [31, 32]. The ${\beta}_{\mathrm{RC}}$ measures the extent to which the actual communities deviate from the null (stochastic) model simulations: a value approaching −1 or 1 means that observed differences in actual communities are lower or higher than null model simulations, referring to deterministic factors favoring similar or dissimilar communities, respectively [31, 32]. The permutational analysis of multivariate dispersions (PERMDISP) was adopted to determine if ${\beta}_{\mathrm{RC}}$ was significantly different between actual communities and null model simulations across each time point under different health conditions [33]. The PERMIDISP compared the distance of the centroid of each group (actual microbial communities vs. null models) and if the *P*-value of PERMIDISP is <.05 (as a significance), it suggests that actual microbial communities are significantly different from null models, indicating more deterministic-driven community assembly [33]. Furthermore, the modified stochasticity ratio (MST) was used to quantify the stochasticity of microbial community assembly in R (“NST” package) [9]. This metric ranges from 0 to 1 with 0.5 as the boundary point between more deterministic (<0.5) and more stochastic (>0.5) assembly [9].

### Interactions between microbial assembly patterns and microbial community structures

First, we evaluated if assembly patterns related to gut microbial compositions and diversities regardless of microbial perturbations and yeast supplementation. The partial correlation [34] was adopted to correlate the relative abundance of a taxon (at phylum level) and SES in R (“ppcor” package) [35]. In addition, the Dirichlet multinomial model (DMM) in R (“Dirichlet Multinomial” package) [36, 37], which clusters samples based on similarities of microbial community structures, was used to identify key stages of microbiota development at the genus level. The number of key stages was determined by the lowest Laplace approximation score, and the top 10 genera with the highest assignment strengths were considered the main drivers for microbial community structure at each stage [36]. Linear models were constructed to determine associations between alpha diversities and SES across ages.

Microbial networks were constructed between CON-H and CON-UH across ages at the genus level using Spearman’s coefficient (Spearman’s *R* > 0.6, *P* < 0.05) and topological properties, including modularity (value > 0.4, suggesting that the network has a modular structure) [38], average degree (the average number of connections per node) [39], and clustering coefficient (the degree to which nodes tend to cluster together) [40] computed for each network. Then, two topological properties (average degree, clustering coefficient) representing node distributions were pairwise compared across ages in both CON-H and CON-UH using nonparametric Kolmogorov–Smirnov (KS) tests. In addition, critical genera were identified from networks using the integrated value of influence (IVI) ranging from 0 to 1, with 1 representing the most influential nodes across the whole network in R (“influential” package) [41]. For each constructed network, only the top 10% of the most influential genera were presented. Furthermore, linear mixed models were adopted to determine if alpha diversities (fix effect) were associated with MST representing stochasticity under different health conditions with groups (CON-H and CON-UH) and ages as random effects.

### Modeling of diarrhea–probiotic interactions affecting transitions of assembly patterns

The two-state Markov model in R (“msm” package) [42] was used to identify conversions of assembly patterns between two states (either stochastic or deterministic) in longitudinal observations given the existence of certain covariates (diarrhea and probiotic supplementation in our study). The two-state Markov model was constructed by subgrouping samples by their diet (Placebo/SCB) and defining two interconversion states. Here, forward transitions referred to transitions from stochastic- to deterministic-driven assembly and vice versa for backward transitions. The transition probabilities between the two states were calculated, and associations with the covariate (host health condition: healthy/scour) were quantified. In each case, the relationship between state transitions and the covariate was defined as hazard ratio (HR) with a 95% confidence interval (95% CI).

### Identification of microbial ecotypes for assembly patterns in response to diarrhea and SCB supplementation

To quantify microbial ecotypes in microbial communities, the niche breadth index that measures the diversity of resources used by an individual was adopted as follows:


\begin{equation*} {B}_j=\frac{1}{\sum_{i=1}^N{P}_{ij}^2}. \end{equation*}

Here, ${B}_j$ stands for niche breadth, ${P}_{ij}$ refers to the proportion of any species $i$ in a given sample, and $j$ and $N$ are the total number of samples [43]. Based on the 1000-time simulations (quasiswap permutation algorithms, EcolUtils R package), three microbial ecotypes were identified: the empirical niche breadth value of a certain taxon that exceeded the upper limit of the 95% confidence interval of the null distribution was designated as a generalist (in dominant positions with a wider ecological niche), the lower limit of the 95% confidence interval was defined as a specialist (harboring a narrow niche breadth with restricted access to recourses), and within the 95% confidence interval was defined as a neutralist [44].

Furthermore, partial least squares path modeling (PLS-PM) in R (“plspm” package) was used to determine the contributions of predicting microbes to microbial assembly patterns and impacts of other variables (i.e. alpha diversity, age of calves, microbial compositions, host health conditions). In particular, the average relative abundance of microbial ecotypes (generalists, specialists, neutralists), age of calves in days, host health conditions, and assembly patterns determined by Raup–Crick distance were included as observable variables. The alpha diversity that was not directly observed but could be interpreted by Shannon and Chao1 indices was considered the latent variable. The path coefficients were utilized to assess interactions between two variables with positive/negative path coefficients, representing positive/negative interactions [45].

### Identification of bacterial markers predicting assembly patterns

The Boruta method was adopted to identify predictive markers for assembly patterns using the “Boruta” package in R [46, 47], of which the presence/absence of bacterial taxa was used for assessing the relative importance of each microbe being predictive for assembly in SCH-H and SCH-UH, respectively, with age (from w1 to w8) as a confounding feature. To ensure that selected microbes were ecologically important, markers that were designated to generalists/specialists were included for analysis. The accuracy of bacterial markers for predicting assembly patterns was further assessed using the area under the receiver operating characteristic (ROC) curve in R (roc. Curve package) with the standard being excellent (0.9–1.0), good (0.8–0.9), fair (0.7–0.8), weak (0.6–0.7), or fail (0.5–0.6) [46, 48].

## Results

### Microbial diversity and composition were associated with assembly patterns in the gut of neonatal dairy calves

**Figure 1:**
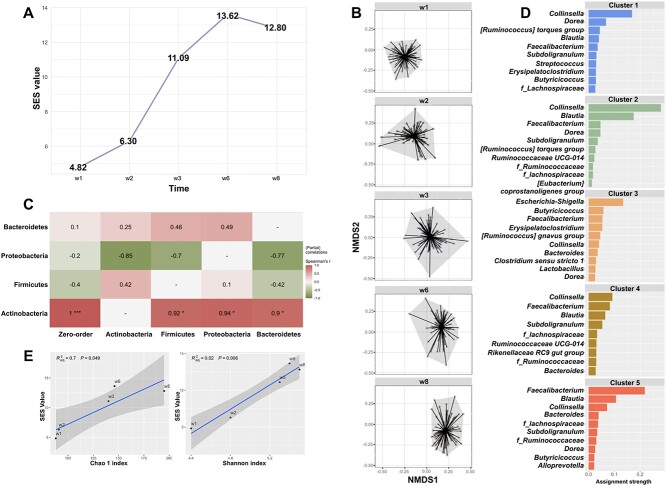
Stochasticity-driven gut microbial community assembly relates to compositions of microbial taxa at phylum and genus level in calves; (A) the SES computed from C-score metrics using null model simulations. The higher SES value stands for the trend of being deterministic-driven assembly; (B) the ordination of community compositions by nonmetric multidimensional scaling based on the Raup–Crick dissimilarity distance; (C) the correlations between SES and bacterial phyla compositions using the spearman partial correlation with a color gradient representing partial Spearman’s correlation coefficients (^*^P < .05, ^**^P < .01, ^***^P < .001). The “zero-order” column represents Spearman correlations without partial control (that is correlations between the SES value and one certain phylum were constructed without excluding the potential confounding effect of other phylum), and (D) the top 10 genera in each cluster with high assignment strength were identified as drivers for each cluster using Dirichlet multinomial mixture models, and taxa name starting with *f*_ indicates the family level of this identified microorganism, and (E) the relations between alpha diversity and SES values using linear regression models.

The microbial assembly patterns were analyzed using 393 fecal samples collected from 84 preweaned calves during the first 8 weeks of life (Fig. S1, see online supplementary material for a colour version of this figure). The microbial community at w1 and w2 had lower SES values (Fig. 1A) and insignificant distances to null model simulations (Table S1), indicating more stochasticity in these weeks than in the later weeks. However, the microbial community at w3 and w6 had a higher SES value (Fig. 1A) and significant distances to null model simulations (Table S1), indicating more determinism in these weeks. The microbial community at w8 had a higher SES value (Fig. 1A) but insignificant distances to null model simulations (Table S1). Besides, microbial structures were highly dissimilar at w1 and w2 when compared with the later weeks based on the significant pairwise differences in ${\beta}_{\mathrm{RC}}$ (Fig. 1B, Table S2).

The partial correlation analysis revealed that the abundance of the Actinobacteria phylum was positively correlated with deterministic-driven assembly (*P* < .01, Fig. 1C). The DMM model revealed that five clusters based on the similarity of microbial profiles at the genus level (Cluster 1: *n* = 113, Cluster 2: *n* = 87, Cluster 3: *n* = 78, Cluster 4: *n* = 64, Cluster 5: *n* = 51, Fig. S2A, see online supplementary material for a colour version of this figure). A higher proportion of samples collected from the same time point were grouped into the same cluster; Cluster 3 (70 out of 78, 90%), Cluster 5 (69 out of 113, 61%), Cluster 1 (44 out of 51, 86%), Cluster 2 (52 out of 87, 60%), and Cluster 4 (42 out of 64, 65%) represented the microbial profiles for w1, w2, w3, w6, and w8, respectively (Fig. S2B, see online supplementary material for a colour version of this figure). The top 10 genera were identified from each cluster (Fig. 1D), and *Escherichia-Shigella* from the Proteobacteria phylum and *Faecalibacterium* from the Firmicutes phylum contributed the most to Clusters 3 (w1) and 5 (w3), respectively (Fig. 1D), while *Collinsella* from Actinobacteria was the most contributing genus for Clusters 1 (w2), 2 (w6), and 4 (w8, Fig. 1D). Moreover, both Chao1 and Shannon indices of the five clusters increased with age (Fig. S2C, see online supplementary material for a colour version of this figure) and were positively correlated with SES values as revealed by the linear regression model (${R}_{\mathrm{Chao}1}^2=0.7,P=.05;{R}_{\mathrm{Shannon}}^2=0.92,P<.01$, Fig. 1E).

### Microbiome assembly patterns differed between healthy and diarrheic calves

Although alpha diversities (both Chao1 and Shannon indices) were significantly increased across ages in both CON-H and CON-UH, a higher Chao1 index was observed in CON-H at w2 and w6 compared with that in CON-UH (all *P* < .05, Fig. S3A, see online supplementary material for a colour version of this figure). The average MST value ranged from 0.33 to 0.61 in healthy calves and 0.26 to 0.52 in diarrheic calves (Table S3). The increased microbial richness (Chao1 index) led to significantly lower MST values independent of diarrhea and ages (${P}_{\mathrm{null}\ \mathrm{model}\ \mathrm{vs}.\mathrm{full}\ \mathrm{model}}$ < .01, Fig. S3B, see online supplementary material for a colour version of this figure). However, the Shannon index was not related to assembly patterns even after excluding diarrhea and age effects (${P}_{\mathrm{null}\ \mathrm{model}\ \mathrm{vs}.\mathrm{full}\ \mathrm{model}}$ = .96, Fig. S3B, see online supplementary material for a colour version of this figure).

Deterministic-driven assembly was identified for fecal microbial communities in CON across ages (Table 1), while such pattern was masked by different host health conditions. Particularly, among CON-H, only microbial communities at w2 and w3 of ages showed a tendency for deterministic-driven assembly, while stochastic-driven community assembly was observed for fecal microbial communities at w1, w6, and w8 of age (Table 2). However, deterministic-driven assembly was observed for fecal microbial communities at w6 and w8, and a tendency for deterministic-driven was observed at w1, w2, and w3 in CON-UH (Table 2).

**Table 1:** The distance differences of centroids between the true microbial community and null model simulations across time between SCB-fed and placebo-fed calves using PERMDISP.

Week	SCB group				Placebo group			
	Centroid of actual communities	Centroid of null model	*F*	*P*	Centroid of actual communities	Centroid of null model	*F*	*P*
w1	0.02	0.01	0.89	.35	0.03	0.01	5.80	.02^*^
w2	0.01	0.004	4.41	.04^*^	0.03	0.01	3.63	.05 ^*^
w3	0.02	<0.001	7.11	<.01^*^	0.02	0.001	5.52	.02 ^*^
w6	0.004	<0.001	4.80	.03 ^*^	0.06	0.003	14.60	<.01^*^
w8	<0.001	<0.001	5.95	.02 ^*^	<0.001	<0.0001	5.70	.02 ^*^

The *P*-value ≤ .05 (as significant) refers to deterministic-driven assembly.

**Table 2:** The distance differences of centroids between the true microbial community and null model simulations across time in SCB-/placebo-fed health and unhealthy calves using PERMDISP.

Treatment	Week	Healthy group				Unhealthy group			
		Centroid of actual communities	Centroid of null model	*F*	*P*	Centroid of actual communities	Centroid of null model	*F*	*P*
Placebo	w1	0.05	0.02	2.4	.13	0.05	0.02	3.0	.08^‡^
	w2	0.07	0.01	2.9	.1^‡^	0.04	0.01	2.6	.1^‡^
	w3	0.03	0.004	3.7	.07^‡^	0.02	0.007	3.3	.07^‡^
	w6	0.04	0.01	2.4	.13	0.096	0.008	10.7	<.01^**^
	w8	0.04	0.02	0.8	.4	0.004	0.001	3.8	.05^*^
SCB	w1	0.04	0.04	<0.01	.95	0.03	0.01	1.99	.17
	w2	0.02	0.01	3.59	.07^‡^	0.04	0.02	2.12	.15
	w3	0.06	0.007	4.58	.04^*^	0.02	0.0012	4.31	.04^*^
	w6	0.01	<0.001	7.52	.01^**^	0.01	0.0001	4.30	.05^*^
	w8	0.01	0.001	5.93	.02^*^	0.004	<0.001	2.31	.14

The *P*-value ≤ .05 (as significant) stands for deterministic-driven assembly and .05 < *P* ≤ .1.

^‡^As a trend for deterministic-driven assembly.

### Microbial interactions contributed to the different assembly patterns between healthy and diarrheic calves

The constructed co-occurrence networks representing microbial interactions in both CON-H and CON-UH were highly modular (modularity > 0.4), with clustering coefficients ranging from 0.53 to 0.83 in CON-H and 0.61 to 0.80 in CON-UH (Table S4, Fig. S4, see online supplementary material for a colour version of this figure). In particular, remarkably different clustering coefficients were observed across time in CON-H (*P* < .05, Table S5). The significance of clustering coefficients was also identified in CON-UH except for comparisons at ages of w1 versus w2, w3 versus w6, and w3 versus w8 (Table S5). The average degree in CON-H fluctuated significantly across ages (*P* < .01), except for the comparable degrees at w1 versus w3 (Table S5). The average degree in CON-UH stepwise decreased over time from w1 and w2 (8.77, 8.20) to w3 and w6 (6.16, 5.77) to w8 (5.17, Tables S4, S5).

**Figure 2:**
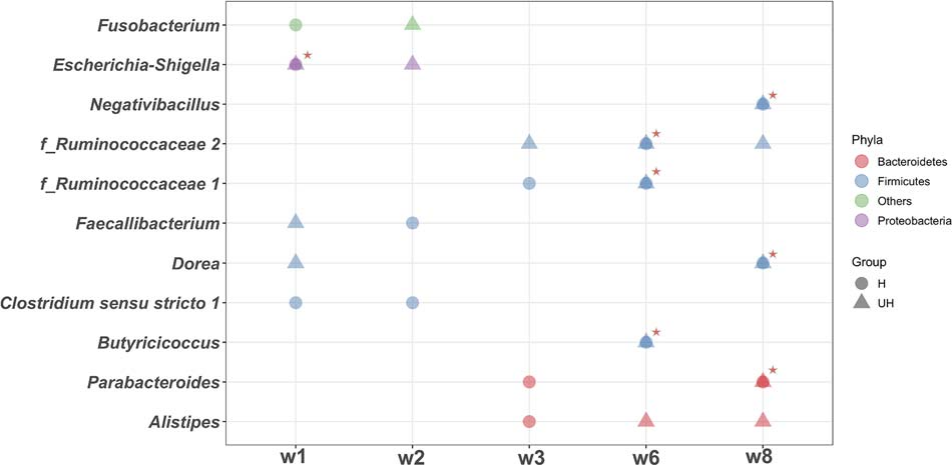
Highly frequent influential genera identified using the IVI approach from microbial interactions in placebo-fed healthy and diarrheic calves. The frequent influential genera stand for any genera that were considered as influential more than once in either healthy or unhealthy groups. The genera with a star refer to the genera simultaneously being influential for both healthy and unhealthy calves.

Furthermore, a total of 25 and 37 influential bacterial taxa were identified from microbial interaction networks in CON-H and CON-UH, respectively (Tables S6, S7). Among these influential taxa, 17 out of 25 and 23 out of 37 genera belonged to the Firmicutes phylum in the CON-H and CON-UH, respectively (Tables S6, S7). Eleven genera (7 from Firmicutes, 2 from Bacteroidetes, 1 from Proteobacteria, and 1 from Fusobacteriota) were found to be influential taxa detected more than once (defined as highly frequent influential taxa in this study, Fig. 2). Among these highly frequent influential taxa, seven taxa were influential taxa for microbial interactions in both CON-H and CON-UH at the same week of age: *Escherichia-Shigella* (w1), and two genera from the families *Ruminococcaceae* (w6) and *Butyricicoccus* (w6), *Dorea* (w8), *Parabacteroides* (w8), *Negativibacillus* (w8, Fig. 2).

### SCB supplementation affected the microbial community assembly shift relative to diarrhea

**Figure 3:**
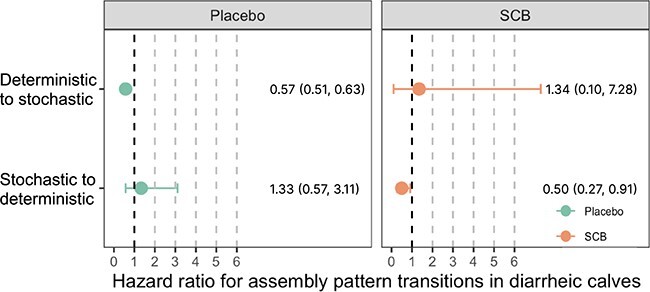
The state transitions in placebo-/SCB-fed diarrheic calves evaluated by the two-state Markov model. The HR greater than 1 (i.e. HR = 1.5) suggests that 50% more likely to support one state transition compared with the control group.

Deterministic-driven assembly was identified for the SCB group across ages except at w1 (Table 1). In SCB-H, assembly patterns were shaped by stochasticity at w1 and by determinism from w2 to w8 (Table 2, Fig. S5, see online supplementary material for a colour version of this figure). In SCB-UH, assembly patterns were driven by stochasticity for w1, w2, and w8, and by determinism at w3 and w6 (Table 2, Fig. S5, see online supplementary material for a colour version of this figure). Correspondingly, the two-state Markov model revealed that the HR for the shift from deterministic to stochastic was 1.34 for SCB-fed diarrheic calves, and therefore, SCB supplementation in diarrheic calves fostered the shift of community assembly pattern from deterministic to stochastic (Fig. 3). However, the HR of the shift from stochastic to deterministic was 1.33 in placebo-fed diarrheic calves, and therefore, diarrhea promotes deterministic-driven assembly in CON-UH (Fig. 3).

### Different microbial ecotypes differentially contributed to microbial community assembly in SCB-responsive and nonresponsive calves

**Figure 4:**
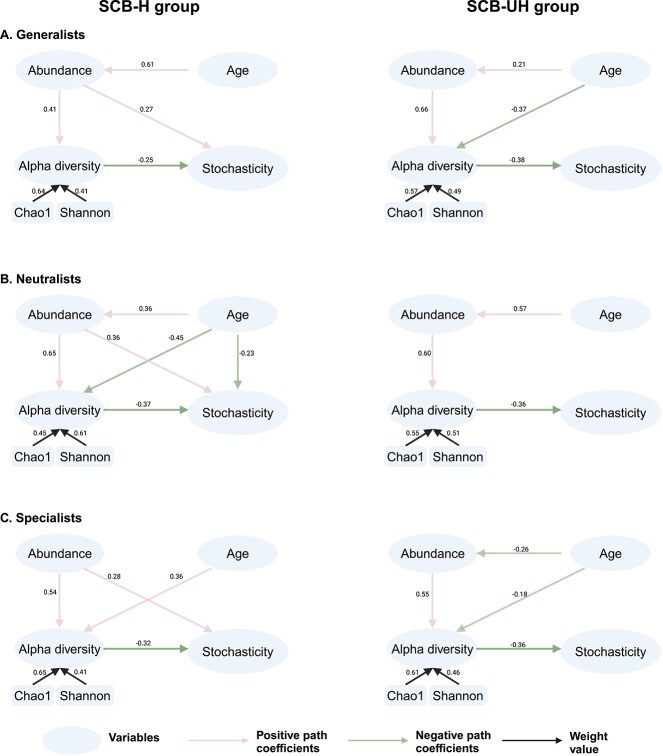
The direct and indirect effects of calf age, the relative abundance of different microbial ecotypes including generalists (A), neutralists (B), specialists (C), and alpha diversities on the stochasticity-driven microbial assembly in SCB-fed healthy (left) or unhealthy (right) calves using the partial least square path modeling. The significant positive and negative relationships were indicated (*P *< .05 as significant). The insignificant relationships are omitted.

An average of 30 $\pm$ 9 specialists and 5 $\pm$ 2 generalists from the SCB-H and an average of 24 $\pm$ 5 specialists and 7 $\pm$ 1 generalists from the SCB-UH (Fig. S6, see online supplementary material for a colour version of this figure). The number of microbial ecotypes identified from each group was not statistically different (Kruskal–Wallis test, both *P* > .05). In addition, the specialists belonged to Firmicutes (95 out of 152, 63%), Bacteroidetes (11 out of 152, 7%), and Proteobacteria (11 out of 152, 7%) in the SCH-H and Firmicutes (75 out of 121, 61%), Bacteroidetes (17 out of 121, 14%), and Actinobacteria (13 out of 121, 10%) in the SCB-UH. The three most predominant phyla to which generalists belonged were Firmicutes (18 out of 23, 78%), Actinobacteria (3 out of 23, 14%), and Bacteroidetes (2 out of 23, 8%) in the SCH-H and Firmicutes (21 out of 33, 63%), Actinobacteria (8 out of 33, 24%), and Bacteroidetes (2 out of 33, 6%) in the SCB-UH. The PLS-PM model further revealed that the abundance of generalists, neutralists, and specialists was positively associated with stochastic-driven assembly in SCB-fed healthy calves, while assembly patterns were not related to the abundance of microbial ecotypes in SCB-fed diarrheic calves (Fig. 4). The stochastic-driven assembly was constrained by increased alpha diversity regardless of microbial ecotypes and host health conditions (Fig. 4).

### Predictive bacterial markers for microbial community assembly pattern differences between SCB-responsive and SCB-nonresponsive calves

**Figure 5:**
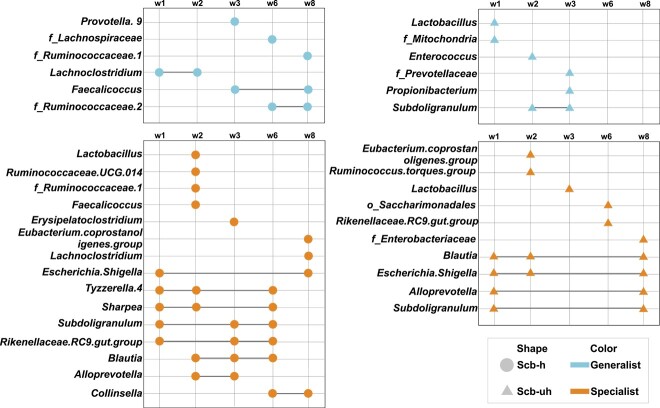
Representative microbial taxa at genus level in both SCB-fed healthy and unhealthy calves. The representative genera were considered as intersections between microbial ecotypes (generalists and specialists) and predicting genera for community assembly identified from the Boruta approach. The microbial generalists and specialists were labeled. The circle or triangle refers to microbes from SCB-H or SCB-UH group, respectively. A genus connected with a line at different time points refers to being a generalist/specialist more than once.

Using the Boruta method, we identified that a total of 23 genera and 21 genera were critical for predicting assembly patterns in SCB-H and SCB-UH, respectively (Table S8). Among the 23 genera in the SCB-H, 6 out of 23 (26%) and 15 out of 23 (65%) microbes were designated as generalists and specialists, respectively (Fig. 5). For identified genera in the SCB-UH, 6 out of 21 (29%) belonged to generalists, and 10 out of 21 (48%) were attributed to specialists (Fig. 5). Interestingly, three generalists (*Lachnoclostridium*, *Faecalicoccus*, and *f_Ruminococcaceae* 2) and eight specialists (*Escherichia*-*Shigella*, *Tyzzerella* 4, *Sharpea*, *Subdoligranulum*, *Rikenellaceae* RC9 gut group, *Blautia*, *Alloprevotella*, *Collinsella*) were classified into their corresponding roles more than once in the SCB-H (Fig. 5). This classification was also observed in the SCB-UH (one generalist: *Subdoligranulum*, four specialists: *Blautia*, *Escherichia*-*Shigella*, *Alloprevotella*, *Subdoligranulum,*Fig. 5). The area under the ROC curve (AUC) approach further confirmed the robustness of bacterial markers identified by both the Boruta method and specialization for predicting deterministic-driven assembly (Figs S7, S8, see online supplementary material for a colour version of these figures). Four out of 18 bacterial taxa (*f_Ruminococcaceae 1*, *Blautia*, *Ruminococcaceae* UCG 014, *f_Ruminococcaceae* 2) and 12 out of 18 markers exhibited good (AUC > 80%) and fair (AUC > 70%) accuracy of predictiveness in the SCB-H group (Fig. S7, see online supplementary material for a colour version of this figure), respectively. In the SCB-UH group, only 5 out of 14 markers existed with fair predictiveness (AUC > 70%) for determinism, and no markers with good accuracy were identified (Fig. S8, see online supplementary material for a colour version of this figure).

## Discussion

Our study showed that neutral theory-based dynamic assembly patterns occurred in the gut microbial community of neonatal calves, providing an advanced understanding of microbial community variations from the assembly perspective in neonatal calves. To date, only one study has reported that neutral-based assembly existed in the rumen microbiome with age and diet as deterministic factors affecting microbiome assembly [14]. In accordance with the finding that age plays a deterministic role in affecting microbial community assembly in the rumen [14], our results also indicate age-specific microbial assembly in the gut with the transition from stochastic at the first 2 weeks of age to deterministic-driven during the age of weeks 3–8. These results suggest that the relative importance of determinism and stochasticity is dynamic, and the age or growth of calves could be a key factor affecting such assembly dynamics. With increased age during early life, calves have boosted host immunity, increased gut barrier integrity [49], altered metabolism and gut physiology [50], and increased microbial diversities [51], which can in turn affect the gut microbiome composition, assembly, and establishment. Our results further revealed that the microbial composition (at the phylum and genus levels) was associated with assembly patterns and such relationship was age-dependent. Similarly, microbial richness (Chao 1 index) and evenness (Shannon index) were positively related to the deterministic processes, which is in accordance with previous findings [52]. Possibly age is the cause of increased richness and evenness which plays a deterministic role during community assembly, suggesting that these host factors (i.e. age/growth) could affect the microbial community assembly patterns.

Furthermore, our findings highlighted that both microbial perturbations (i.e. diarrhea) and modulations (i.e. probiotic yeast SCB administration) can reshape the assembly patterns of the calf gut microbiota. Diarrhea in mammals can trigger gut microbial dysbiosis that leads to compositional shifts and lower diversity [53, 54]. Our results revealed that gut microbial community assembly was more deterministically driven in placebo-fed diarrheic calves, which corresponds to a previous study that showed that the deterministic-driven process was dominant in low-diversity microbial communities [55]. Diarrhea has a detrimental effect on intestinal barrier integrity; as a result, the abundance of pathogenic bacteria was selectively increased [54]. Since microbes can interact with each other with certain microbes driving such interactions, they are considered as keystone taxa [56]. Our results revealed that *Escherichia-Shigella* was a highly frequent influential taxon among microbial interactions. This taxon is an etiological genus responsible for diarrhea, suggesting the critical role of pathogenic commensal bacteria in the pathogenesis of diarrhea. However, the interactions between pathogenic and beneficial bacteria existed in the bovine gut. Specifically, the butyrate-producing bacteria (i.e. *Butyricicoccus* and *Faecallibacterium* [57, 58]) identified from microbial interactions were designated influential genera in placebo-fed healthy and diarrheic calves. Butyrate is a major microbial fermentation product that can induce the differentiation of gut mucosal T regulatory cells (Treg) for the establishment of host gut immunological homeostasis [22, 59], suggesting the potential beneficial role of butyrate-producing bacteria for host gut health maintenance in diarrheic calves. However, these identified beneficial bacteria were inconsistently associated with microbial community assembly patterns. For instance, *Butyricicoccus* was linked to stochastic processes in healthy calves and deterministic-driven microbial community assembly in diarrheic calves at the same age of w5, suggesting that host health is a critical factor affecting microbial community assembly patterns. Whether different species within the same genus contribute to the same microbiome assembly patterns is unknown. Since only fragments of 16S rRNA genes were sequenced, identifying taxa at the species level can be difficult [60]. Further explorations at the species level using a high-resolution sequencing approach are needed to link microbial communities to their assembly patterns. Regardless, the dynamic interplay between influential taxa identified from microbial interactions and host health is likely to reshape microbial communities to accommodate the gut environment, potentially leading to deterministic-driven microbial community assembly in diarrheic calves.

Dietary probiotic administration can restore healthy gut microbiota through several mechanisms, such as intestinal immunomodulation, leading to the prosperity of microbial evenness and diversity and a higher degree of ecological stability [61, 62]. Our study showed that probiotic supplementation led to deterministic-driven microbial community assembly in healthy SCB-fed calves compared with placebo-fed calves, suggesting the beneficial role of probiotics in shaping microbial communities in the gut of healthy dairy calves. As mentioned above, diarrhea is considered as a deterministic factor affecting microbiome assembly patterns in CON-UH calves, which can lower gut microbial diversities. However, the two-state Markov model identified that microbial community assembly patterns tended to transition from deterministic- to stochastic-driven assembly in SCB-fed diarrheic calves. This suggests that diarrhea caused the deterministic-driven assembly in unhealthy calves that could be alleviated by SCB administration. Indeed, our previous study indicated that SCB supplementation can decrease the severity of calf diarrhea [20], and this finding provides potential ecological mechanisms for SCB beneficial effects on the host. However, future verifications are needed to verify this assumption.

Additionally, Vellend [10] unifies niche and neutral theories by considering both determinism and stochasticity into four fundamental processes: diversification (the evolutionary process of generating new genetic variation), selection (ecological factors that alter community structures due to fitness differences), dispersal (movement and establishment of microorganisms across space), and ecological drift (stochastic changes due to random processes of microorganisms birth, death, and reproduction). Among these four processes, dispersal can be much less examined and poorly understood due to the nature of microorganisms (small size, high abundance, and short generation time) [13]. However, the ecological drift was estimated in our study using different mathematical models, and we revealed that stochasticity is critical for gut microbiome assembly. Also, our study identified that microbial perturbation and modification can be a selection force for community assembly that reshapes microbial community structures. Antibiotics were used to treat diarrhea for both CON and SCB calves in this study [22], and the usage of antibiotics is capable of reshaping the gut microbiome [63]. Therefore, whether antibiotic treatments were confounded with SCB feeding and/or diarrhea affecting community assembly patterns, and thereafter, gut microbiome structures need our further exploration. In addition, our study does not identify the genetic mutations of microbes, which could alter a key functional trait(s) that may have a considerable influence on the microbial community [13]. Further whole genome sequencing may be used to assess how diversification affects community assembly.

Our results further revealed that microbial ecotypes are health- and treatment (e.g. SCB/placebo)-dependent and are predictive for microbial assembly patterns, suggesting that microbial perturbations and modulations could affect microbial ecotypes. For instance, one taxon belonging to the *Prevotellaceae* family was the only genus with the highest predicting value for assembly patterns in SCB-UH, while it was not predictive for microbial assembly in SCB-H. However, several microbes, such as *Faecalicoccus*, *Lactobacillus*, and genera from the family *Ruminococcaceae*, were found to be both generalists and specialists in SCB-fed healthy or diarrheic calves at different ages and were identified to be predictive for microbial assembly patterns. Besides, certain predictive microbes for assembly patterns including *Blautia* and *Escherichia*-*Shigella* were identified as the specialist more than once in SCB-fed healthy or diarrheic calves. Microbes can be either residents in the gut (termed drivers) or poor colonizers with a competitive advantage to overcomplete bacterial drivers (termed passengers) [64]. A previous study identified that the family *Enterobacteriaceae*, to which *Escherichia*-*Shigella* belongs, can function as “drivers” under disease conditions and is capable of proinflammatory function and accumulation of unwanted genetic mutations [64]. However, the disease that altered both the microenvironment and microbial communities could facilitate the gradual replacement of bacterial drivers by passengers [64]. Since niche breadth emphasizes the role of microbes in the community featured by the availability of resources, competition between microbes could contribute to different microbial community assembly patterns. During this process, driver–passenger relations could be a factor affecting microbial colonization across ages. However, further studies are required to classify candidate drivers/passengers and their relationship with microbial assembly patterns. Regardless, we identified several microbes (i.e. genera from the family *Ruminococcaceae*, *Blautia* in SCB-H, *Prevotellaceae*, *Blautia*, and *Escherichia*-*Shigella* in SCB-UH) that could be representative for predicting microbial assembly patterns with positive/negative outcomes of SCB treatment and such knowledge is critical for us to develop effective microbial manipulation strategies. When microbes were selected for microbial manipulation purposes, estimating their ecotypes and assembly patterns to determine the outcomes of microbial modulations could be a novel strategy. It is noticeable that our study used only fecal samples from dairy calves, and whether mucosa-attached microbial communities exhibit similar assembly patterns and how host factors (i.e. immunity) are involved in microbial community assembly under different conditions urges future exploration.

## Conclusions

**Figure 6:**
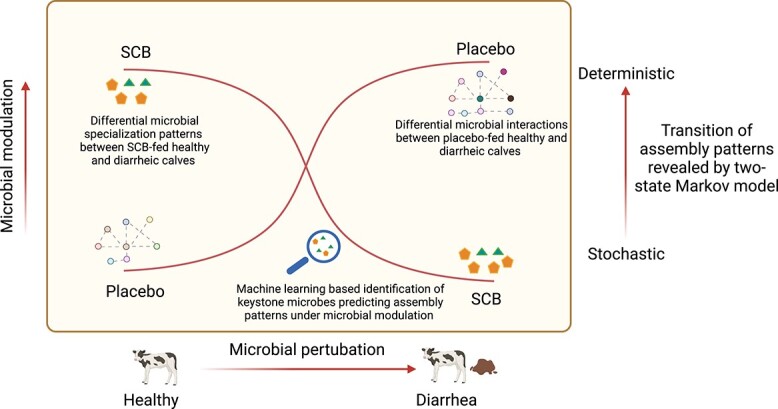
The proposed mechanism of microbial community assembly modulated by microbial perturbation and modulation in neonatal calves. The microbial community of healthy calves is stochastic-driven; however, microbial perturbation (diarrhea) affects microbial interactions and promotes deterministic-driven community assembly. The SCB feeding in healthy calves enhances the deterministic-driven community assembly; however, microbial modulation (SCB feeding) in diarrheic calves promotes the shift from deterministic to stochastic-driven community assembly.

Taken together, our results revealed the existence of neutral theory-based assembly in the gut of neonatal dairy calves, which can be shaped by the deterministic process of both microbial perturbation (diarrhea) and modulation (SCB supplementation). Feeding SCB to diarrheic calves promotes the transition from a deterministic- to stochastic-driven process (Fig. 6). Hence, we propose that the SCB treatment plays a beneficial role in affecting the gut microbiome that can alleviate the negative impact of diarrhea and facilitate stochastic-driven process to reshape microbial communities. Furthermore, several microbes were identified as predictive markers for assembly patterns, highlighting the possibility of manipulating gut microbiota to alter gut microbiome assembly and therefore host health. However, further studies are needed to evaluate our assumptions. Regardless, our results highlight dynamics of microbial community assembly patterns that can be affected by microbial perturbations and probiotic yeast administration with predictive microbes contributing to such process, providing a fundamental understanding of mechanisms affecting microbiome structure and its subsequent interactions with the host during early life. The key taxa affecting the microbiome assembly in the animal gut can be potential targets for microbiome manipulation during early life.

## Supplementary Material

Pan_et_al_Fig_1_ISMECOMMUN-D-24-00077-final_ycae044

Pan_et_al_Fig_2_ISMECOMMUN-D-24-00077-final_ycae044

Pan_et_al_Fig_3_ISMECOMMUN-D-24-00077-final_ycae044

Pan_et_al_Fig_4_ISMECOMMUN-D-24-00077-final_ycae044

Pan_et_al_Fig_5_ISMECOMMUN-D-24-00077-final_ycae044

Pan_et_al_Fig_6_ISMECOMMUN-D-24-00077-final_ycae044

Pan_et_al_Supplementary_file_1_ISMECOMMUN-D-24-00077-final_ycae044

Pan_et_al_Supp_figures_ISMECOMMUN-D-24-00077-final_ycae044

Pan_et_al_Supp_Table_ISMECOMMUN-D-24-00077-final_ycae044

## Data Availability

All DNA sequences were deposited in the NCBI sequence read archive and are accessible under the project numbers PRJNA506828 and PRJNA851017.
