# Supplementary figures and images for: Varied microbial community assembly and specialization patterns driven by early life microbiome perturbation and modulation in young ruminants
Source: ISME Commun. 2024 Apr 9;4(1):ycae044. doi: 10.1093/ismeco/ycae044 (PMC11033733; doi:10.1093/ismeco/ycae044)

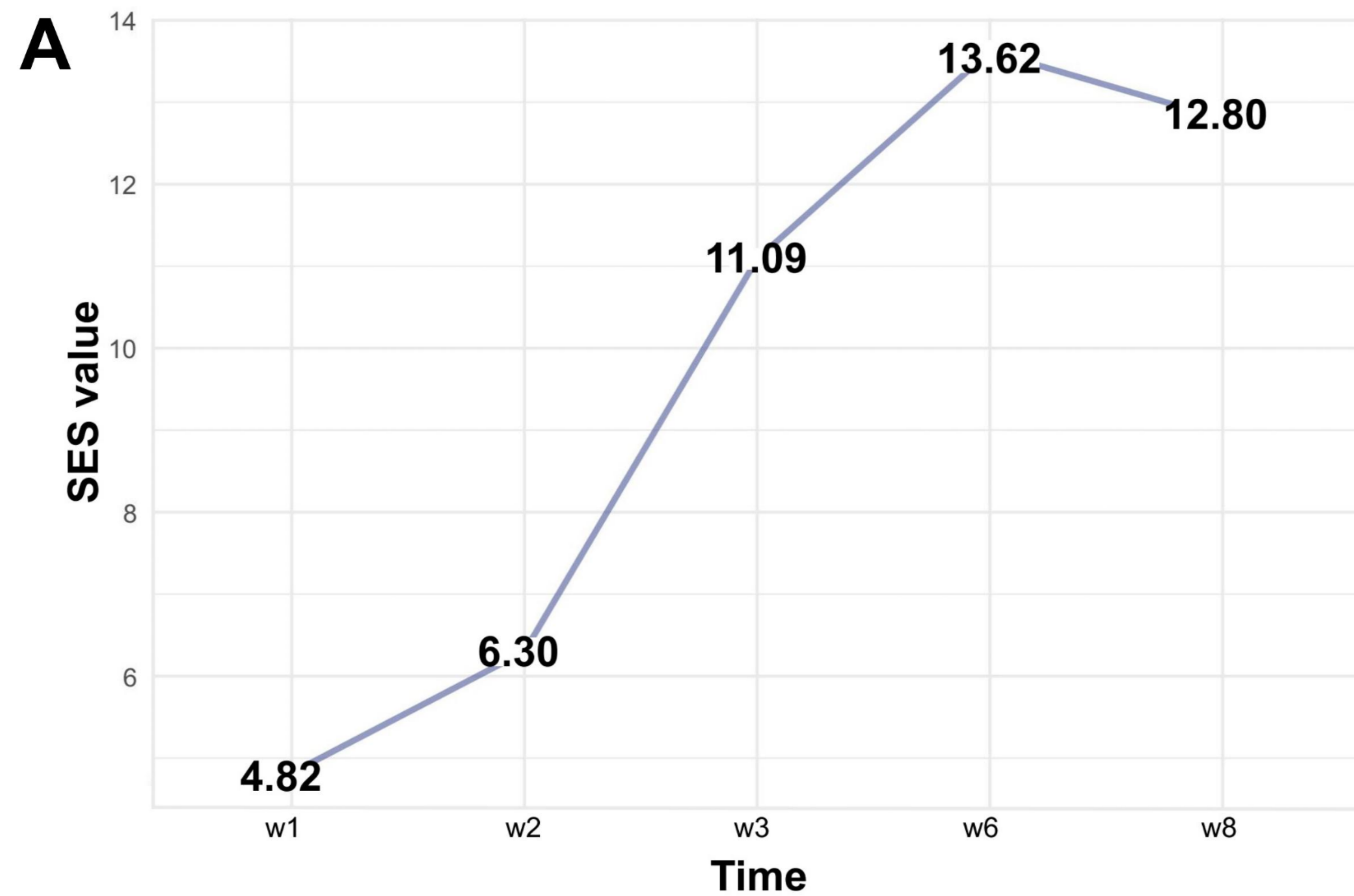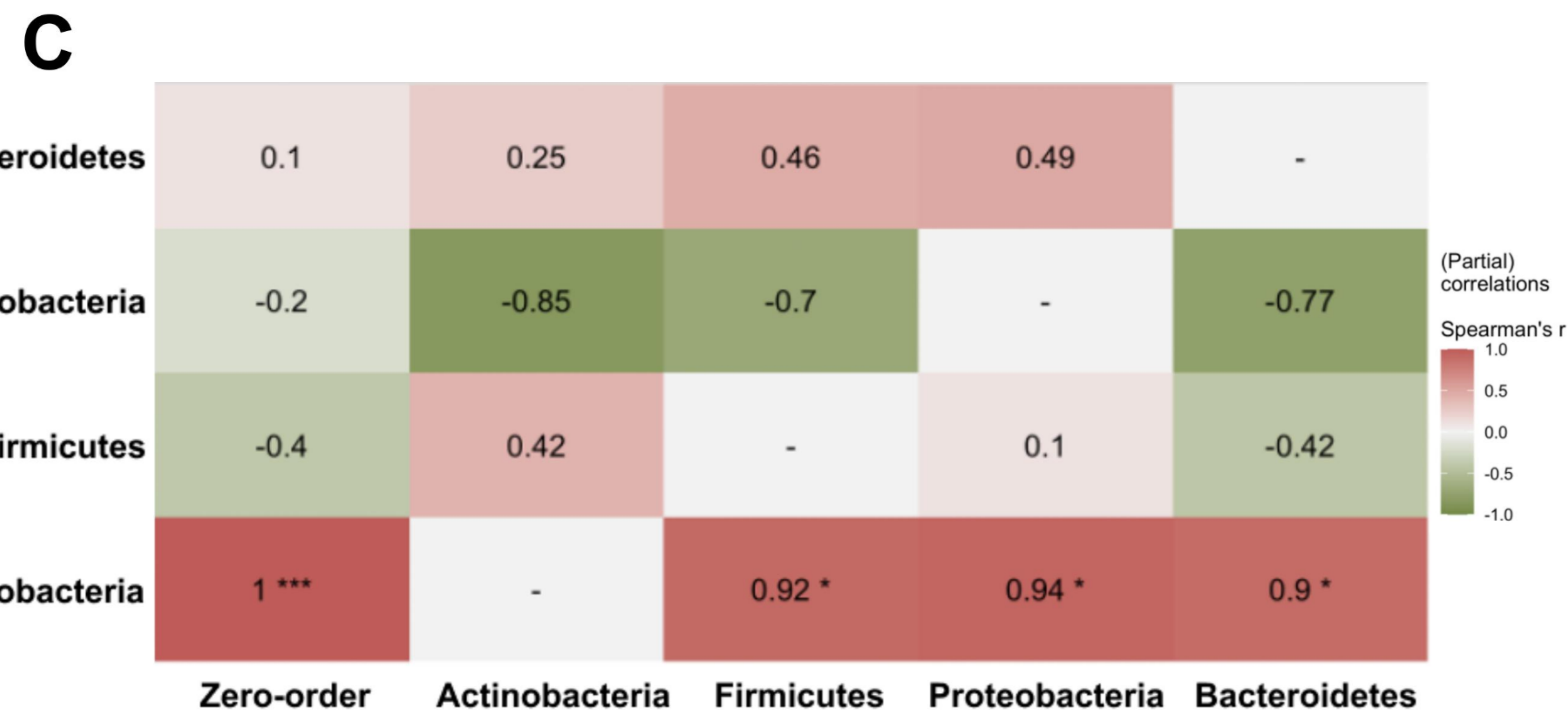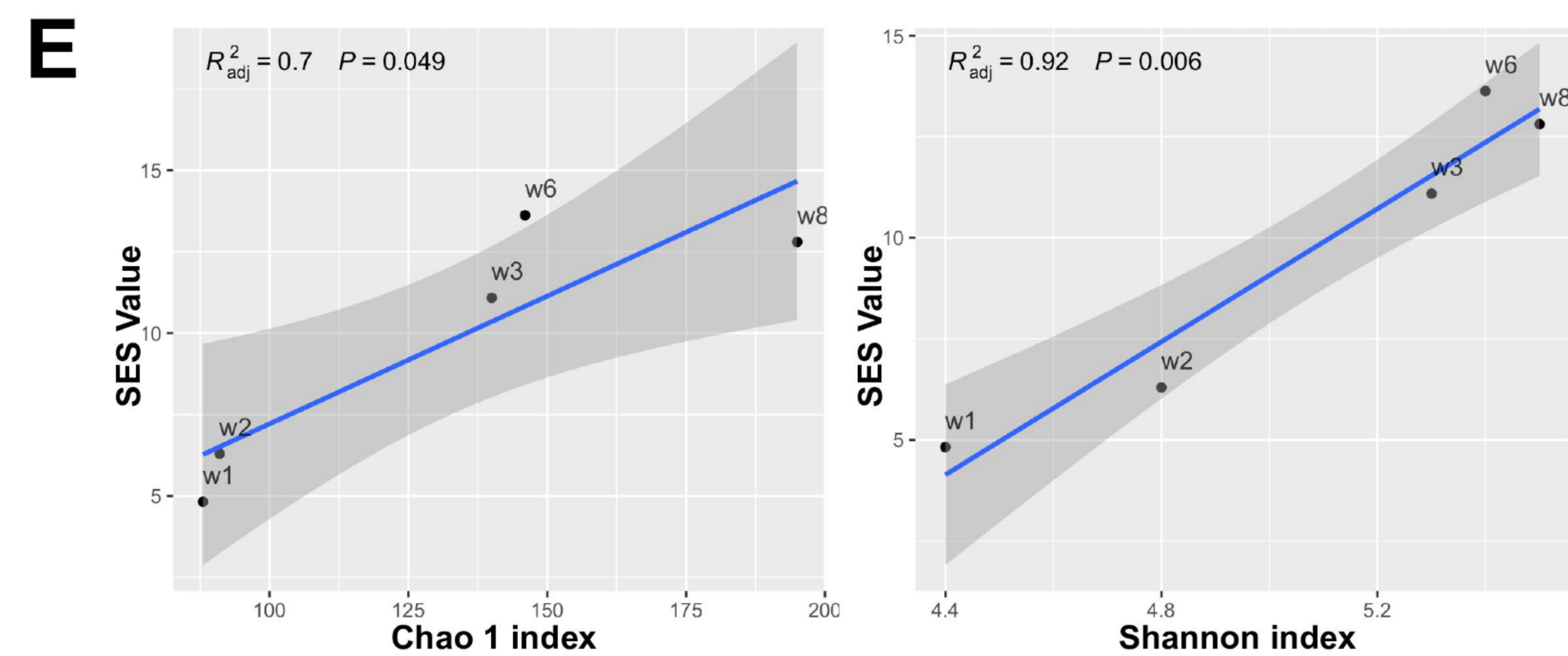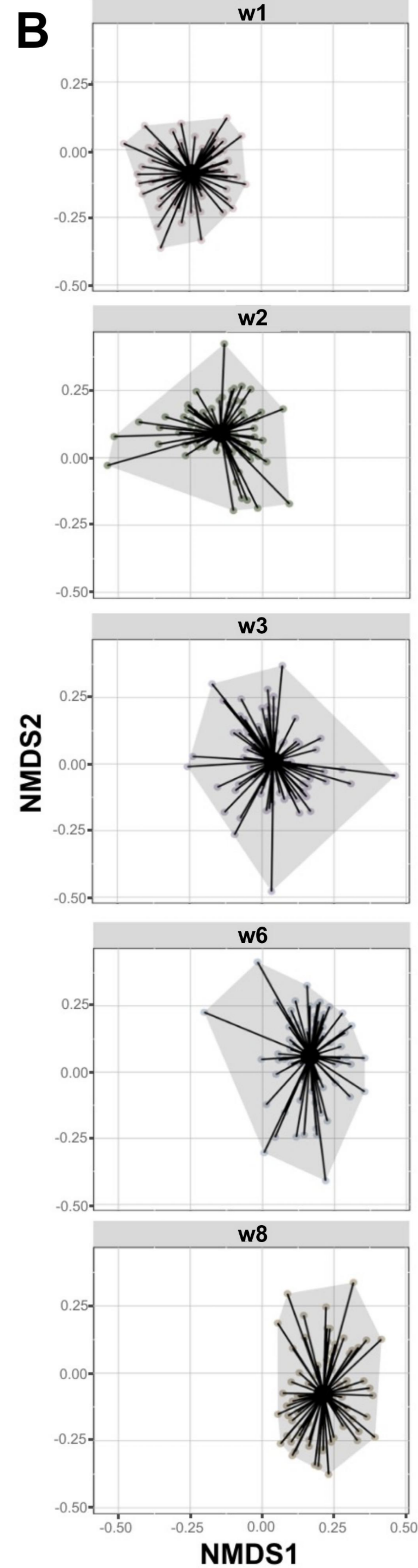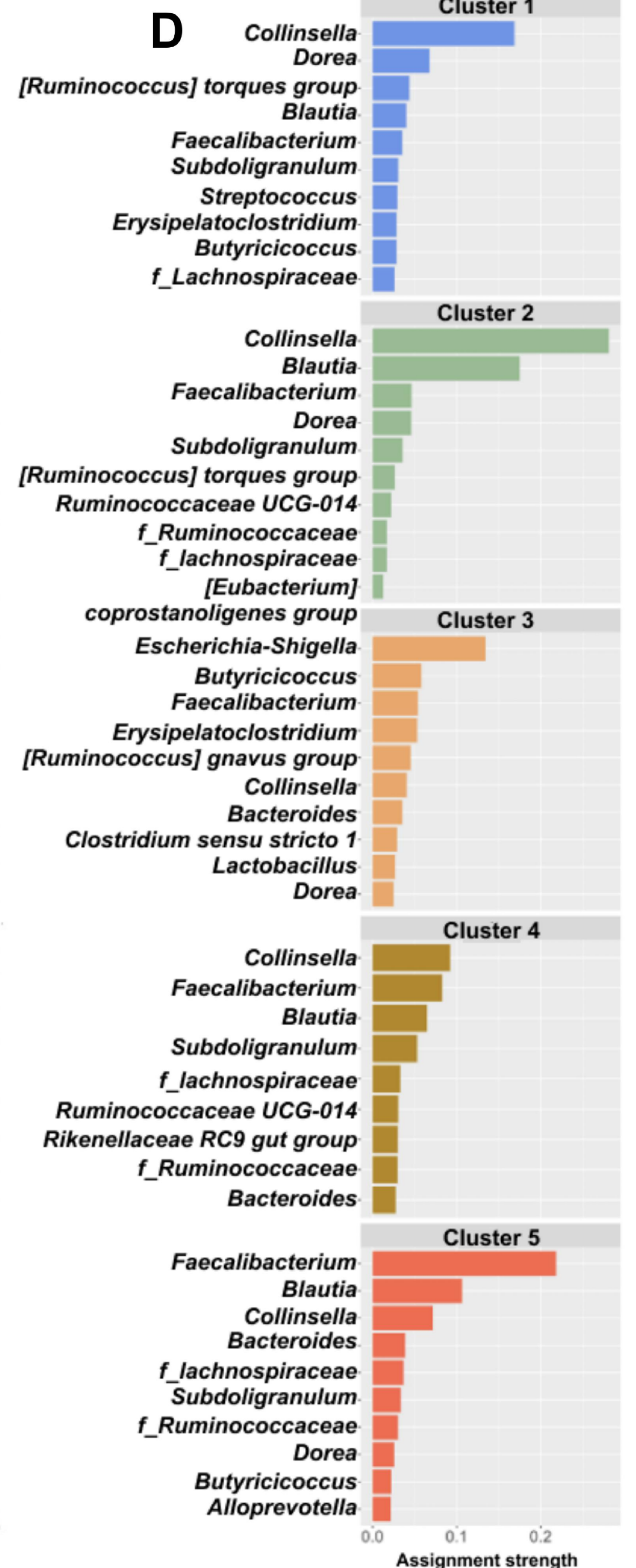

Supplement: Pan_et_al_Fig_1_ISMECOMMUN-D-24-00077-final_ycae044 [file pan_et_al_fig_1_ismecommun-d-24-00077-final_ycae044.pdf]

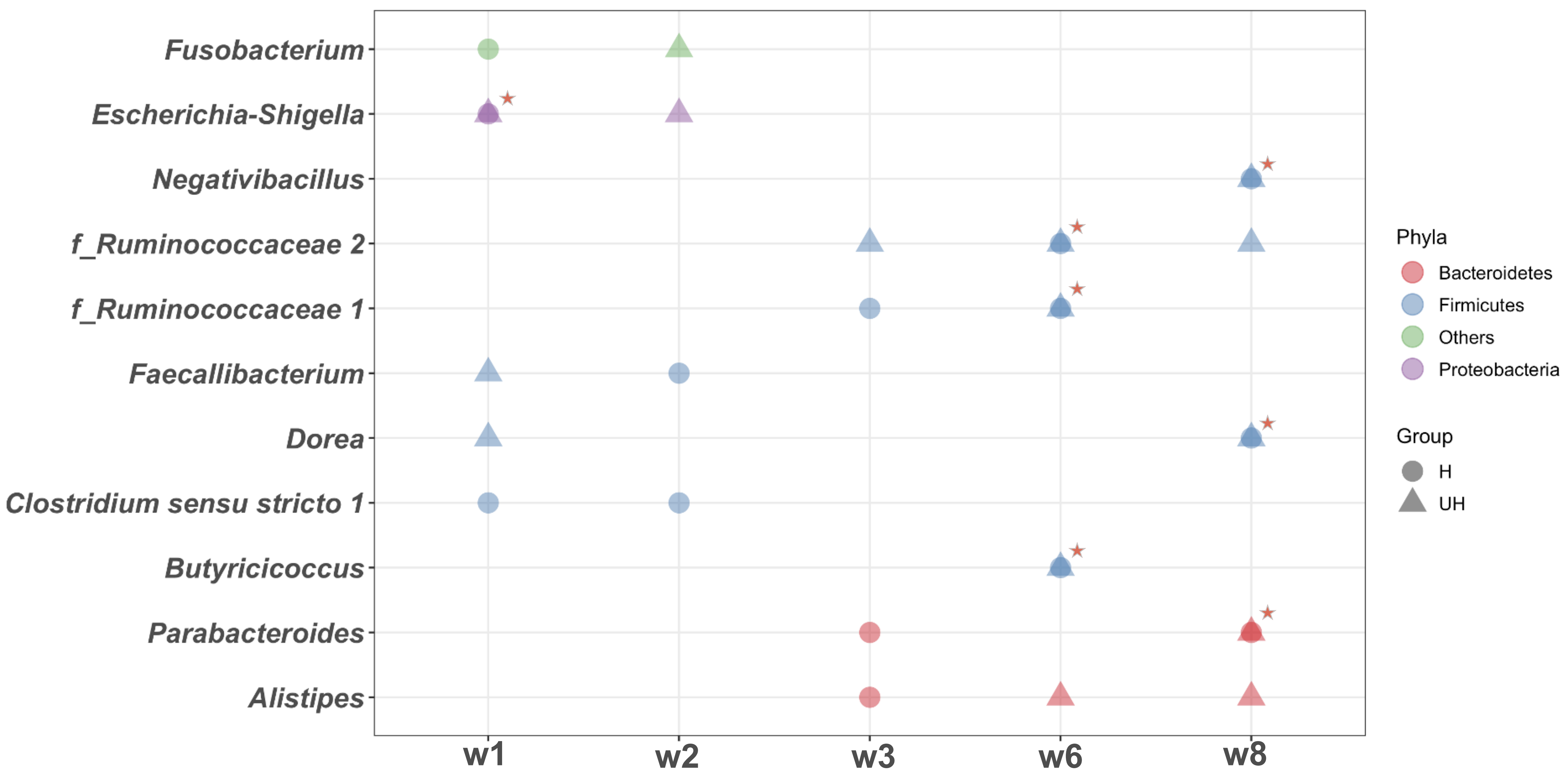

Supplement: Pan_et_al_Fig_2_ISMECOMMUN-D-24-00077-final_ycae044 [file pan_et_al_fig_2_ismecommun-d-24-00077-final_ycae044.pdf]

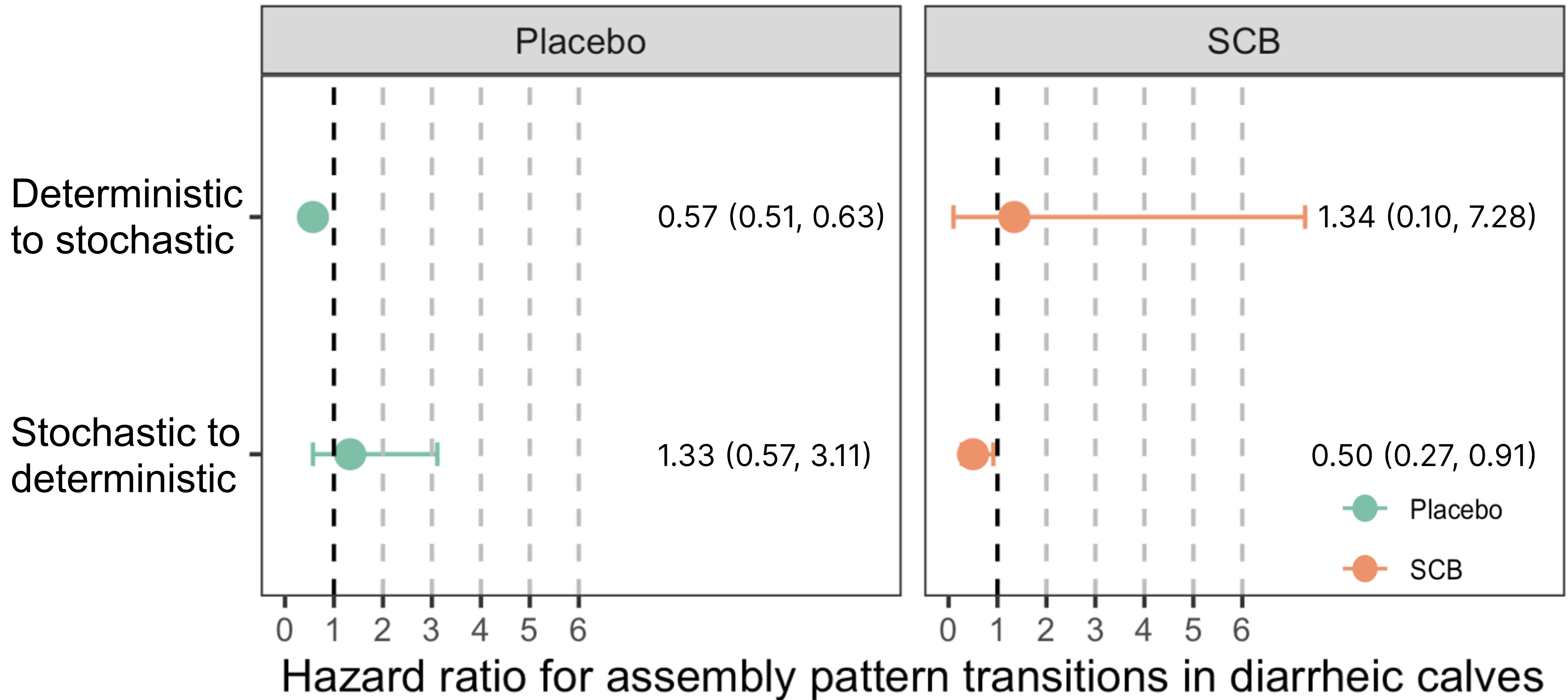

Supplement: Pan_et_al_Fig_3_ISMECOMMUN-D-24-00077-final_ycae044 [file pan_et_al_fig_3_ismecommun-d-24-00077-final_ycae044.pdf]

## SCB-H group

### A. Generalists

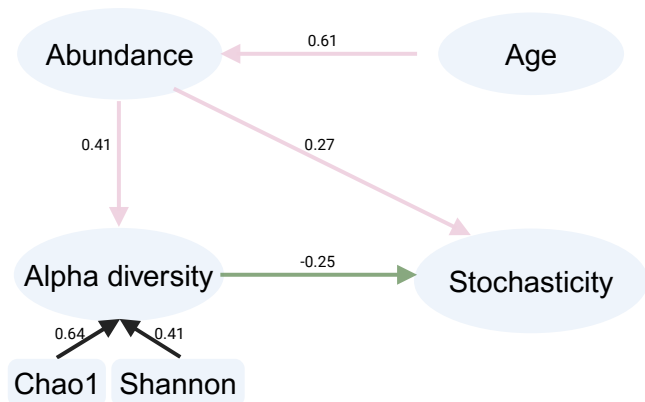

## SCB-UH group

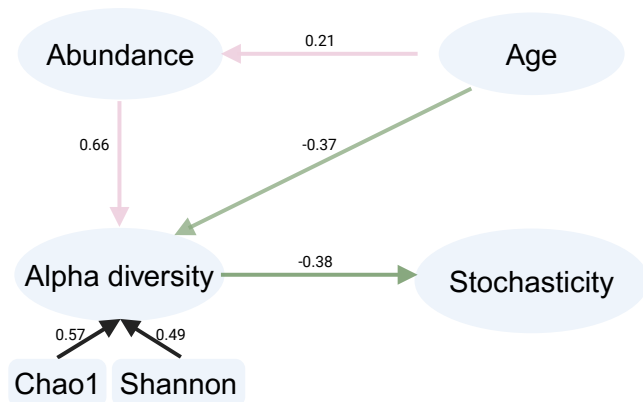

### B. Neutralists

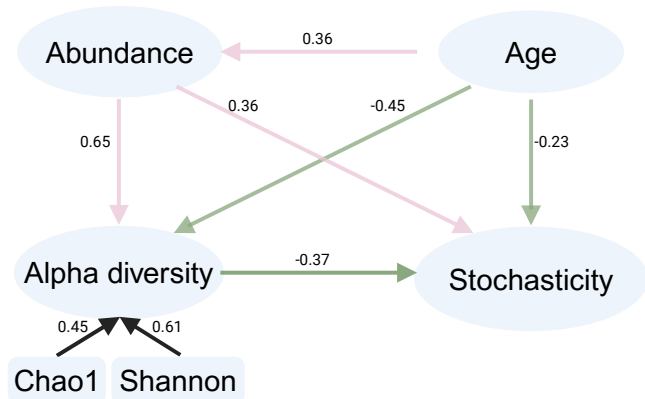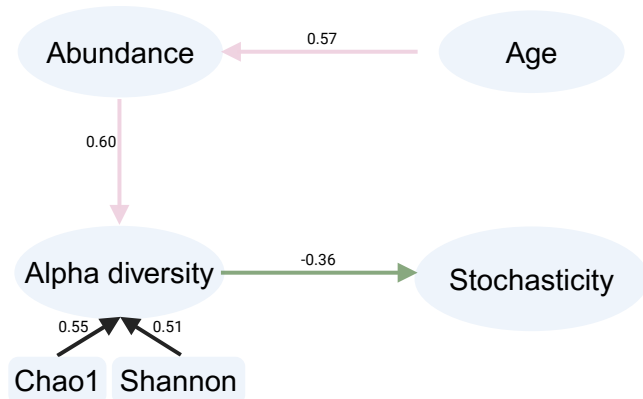

### C. Specialists

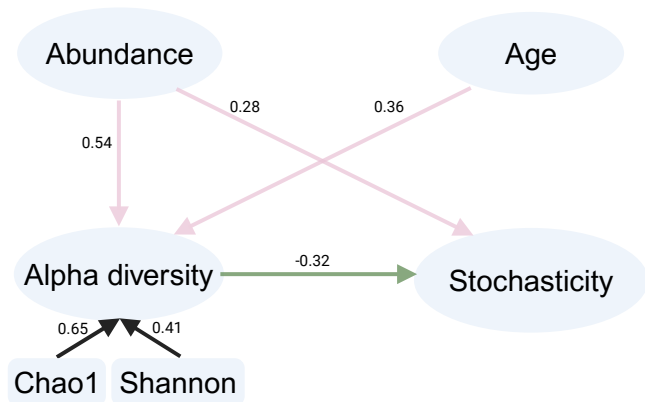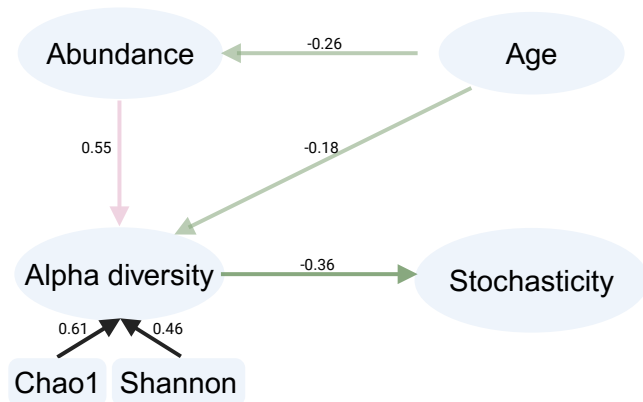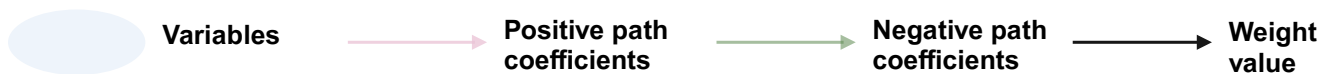

Supplement: Pan_et_al_Fig_4_ISMECOMMUN-D-24-00077-final_ycae044 [file pan_et_al_fig_4_ismecommun-d-24-00077-final_ycae044.pdf]

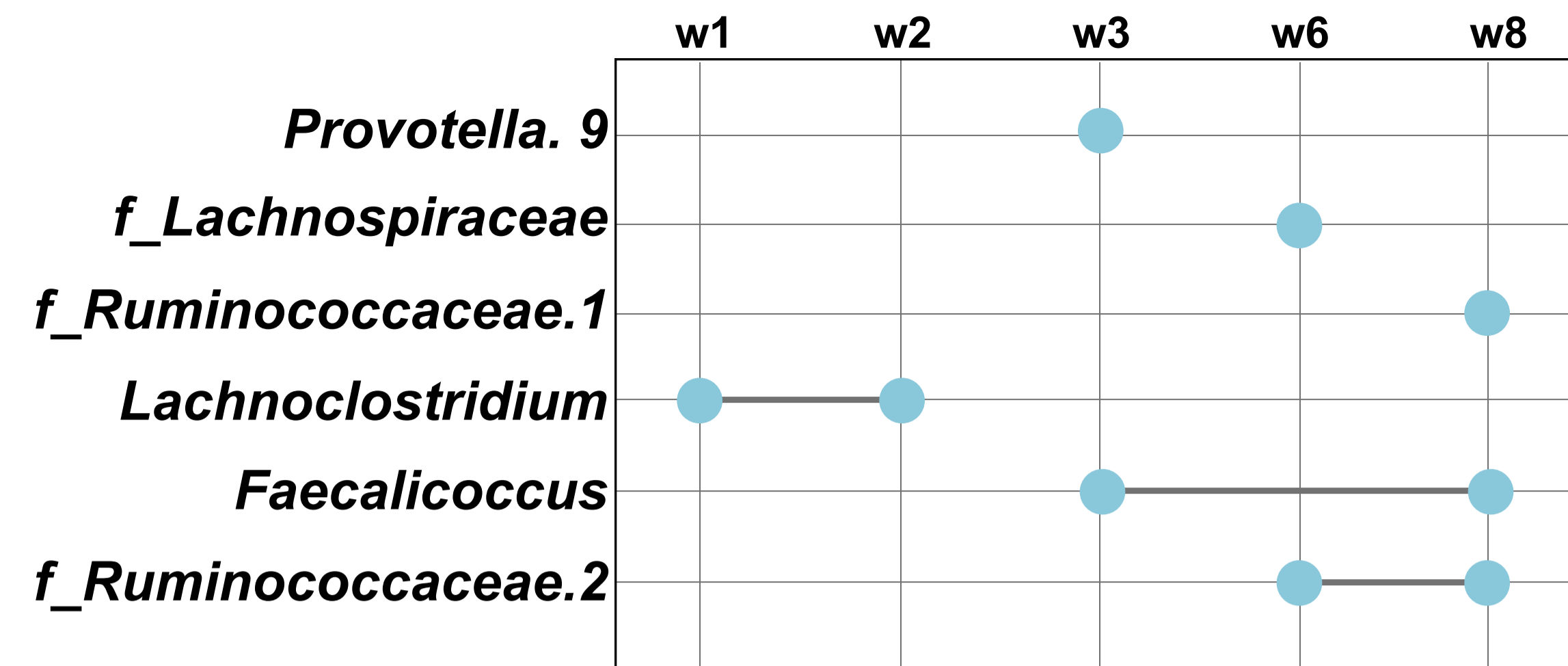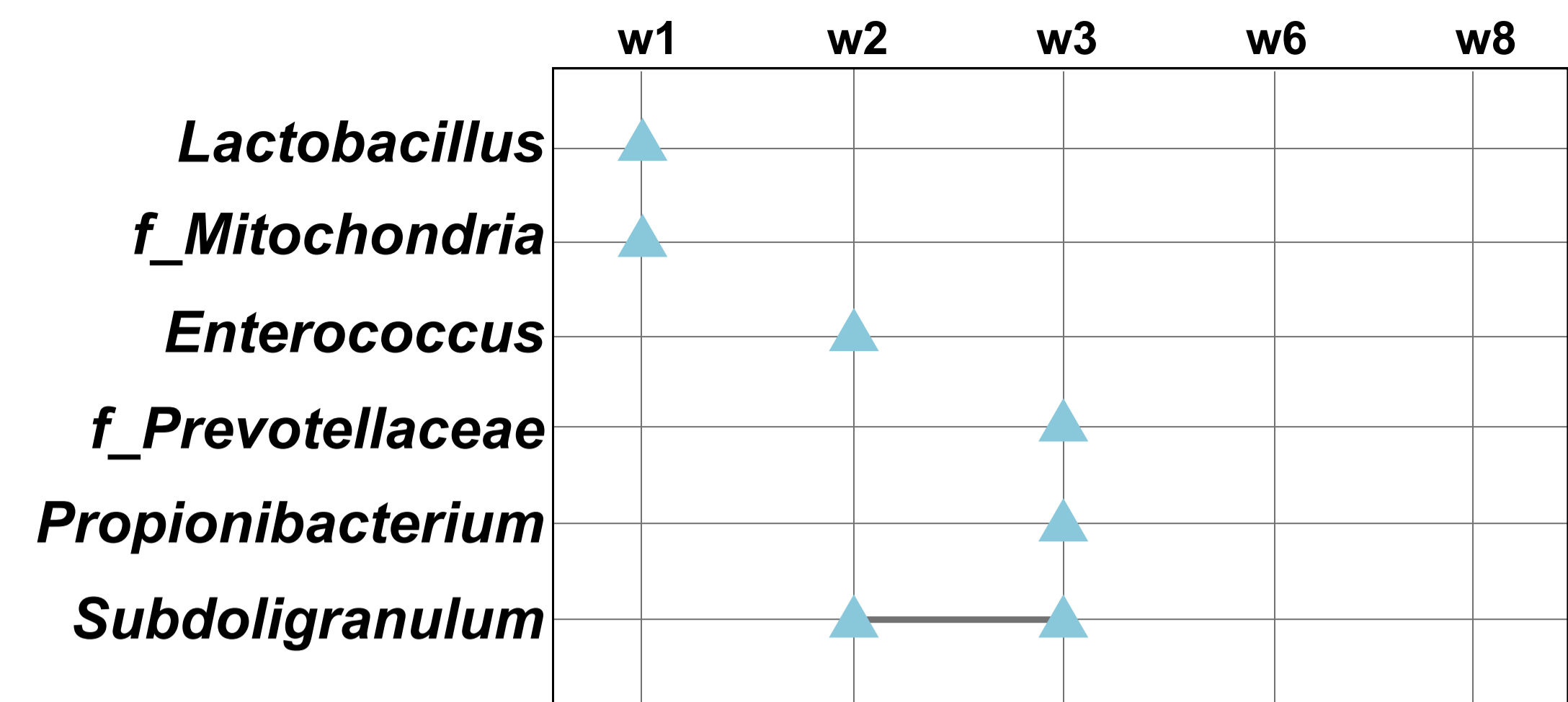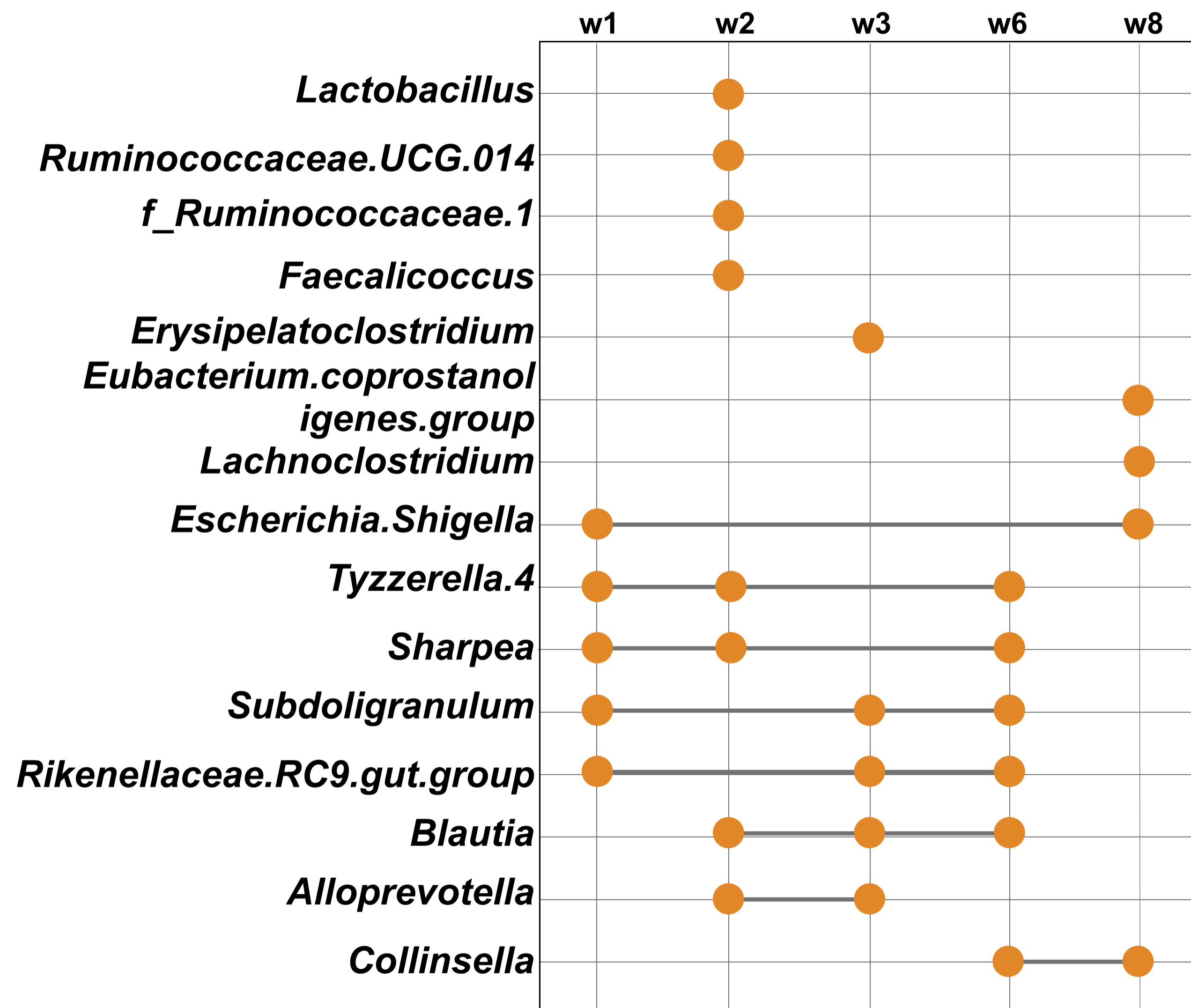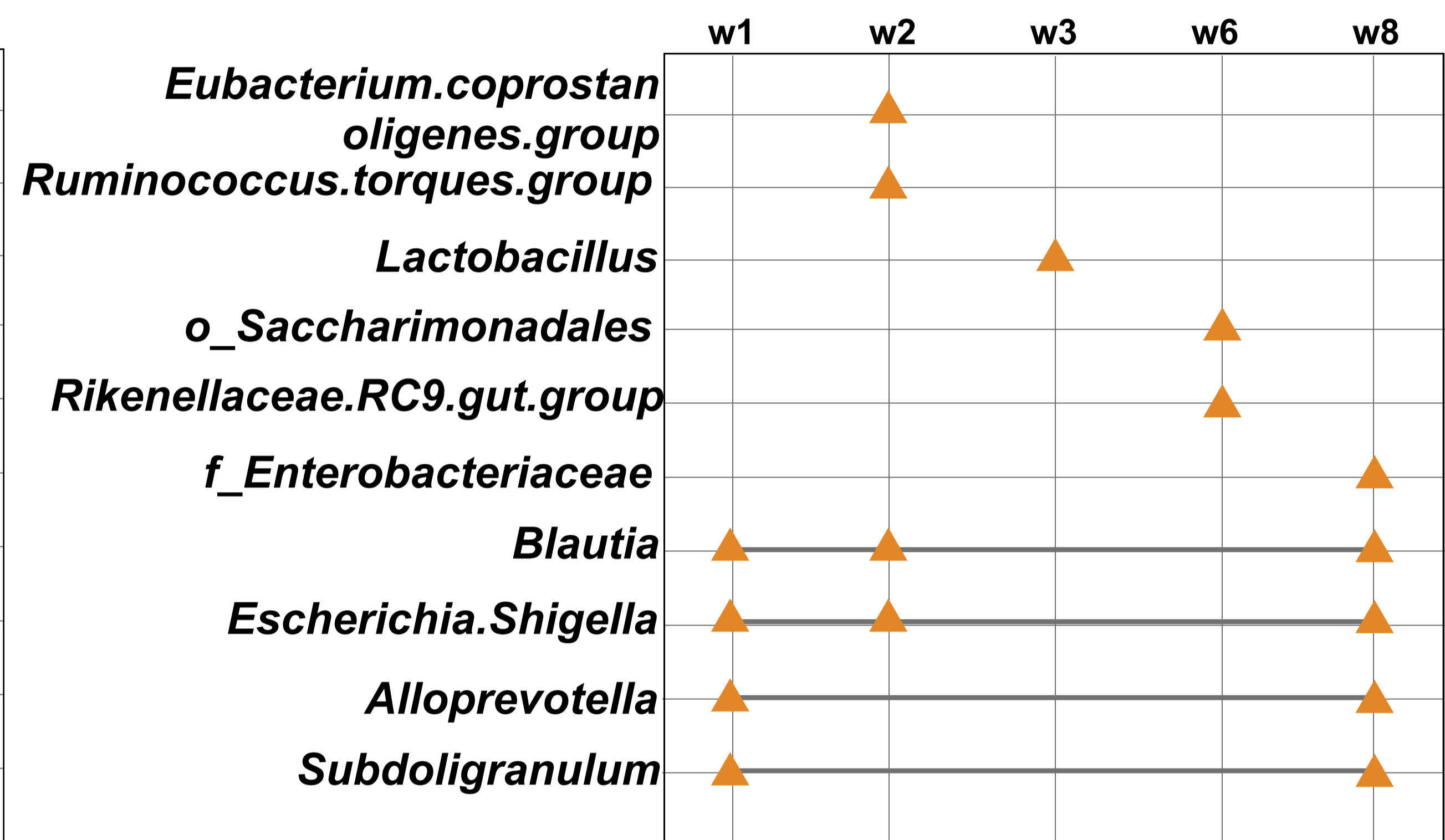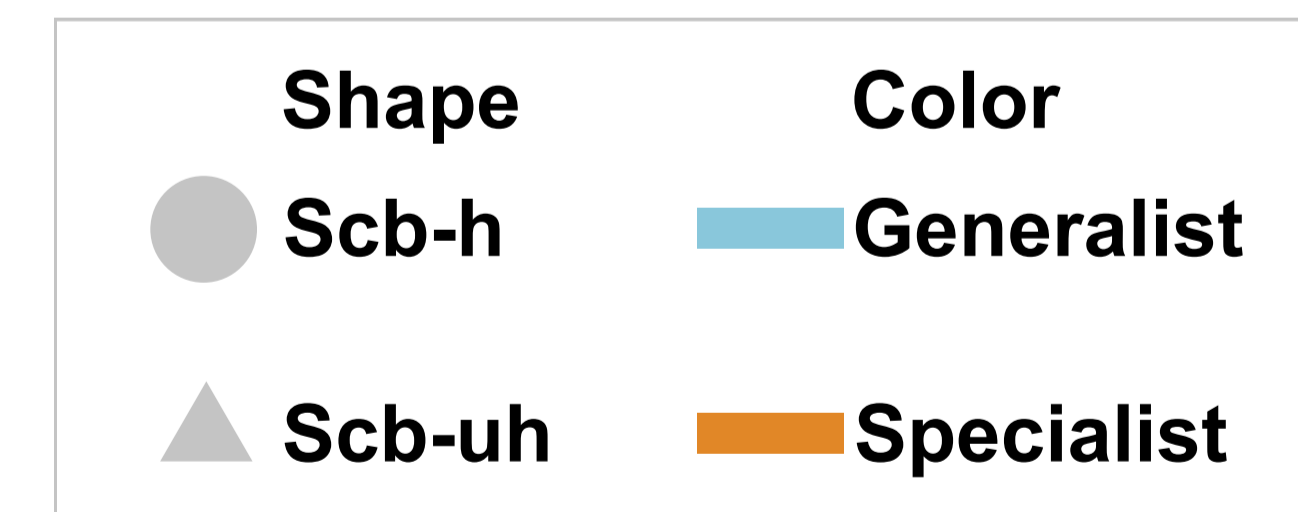

Supplement: Pan_et_al_Fig_5_ISMECOMMUN-D-24-00077-final_ycae044 [file pan_et_al_fig_5_ismecommun-d-24-00077-final_ycae044.pdf]
