# Supplementary material for: Varied microbial community assembly and specialization patterns driven by early life microbiome perturbation and modulation in young ruminants
Source: ISME Commun. 2024 Apr 9;4(1):ycae044. doi: 10.1093/ismeco/ycae044 (PMC11033733; doi:10.1093/ismeco/ycae044)
Supplement: Pan_et_al_Fig_6_ISMECOMMUN-D-24-00077-final_ycae044 [file pan_et_al_fig_6_ismecommun-d-24-00077-final_ycae044.pdf]

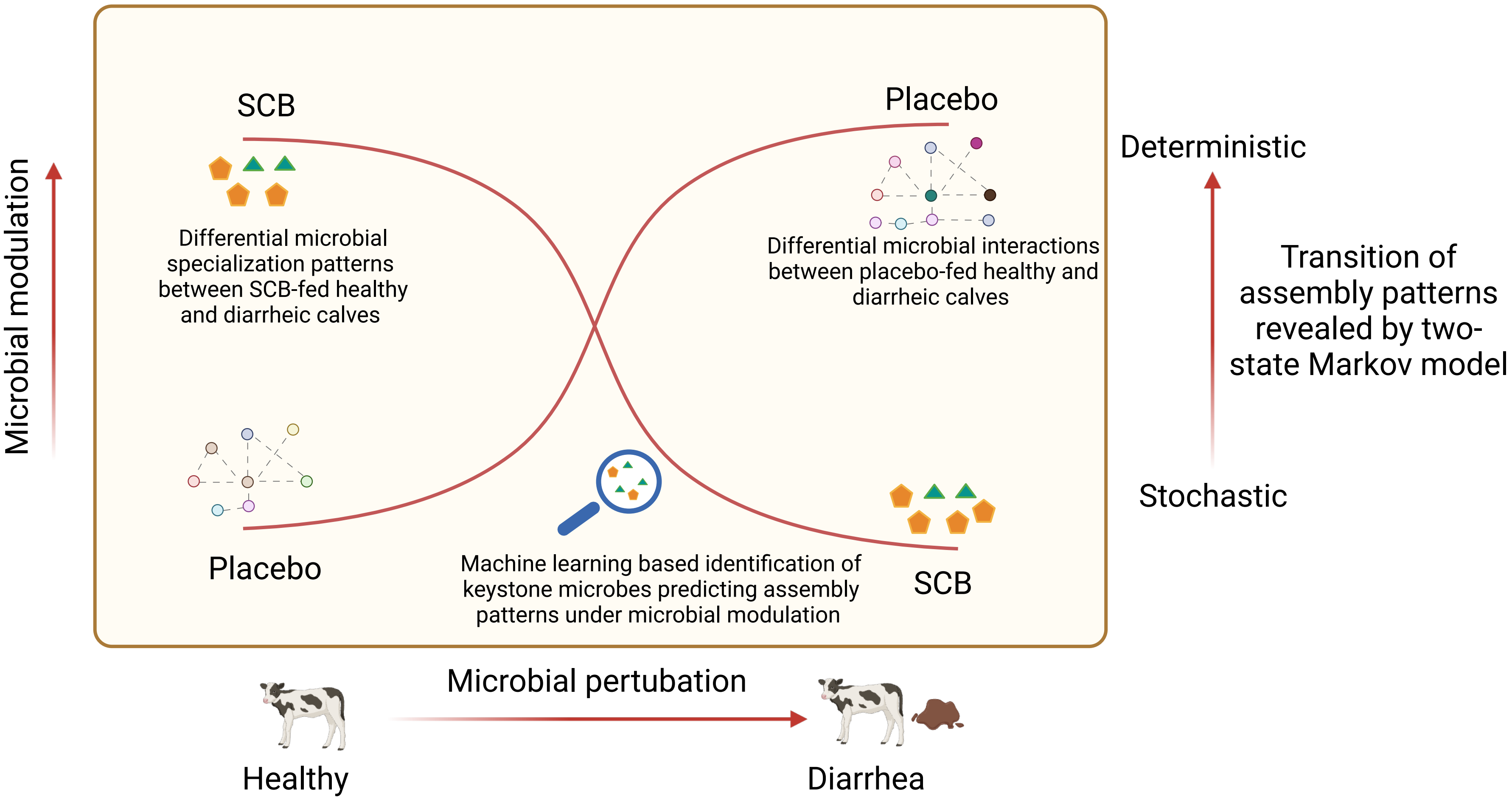
Microbial modulation
SCB
Differential microbial specialization patterns between SCB-fed healthy and diarrheic calves
Placebo
Placebo
Differential microbial interactions between placebo-fed healthy and diarrheic calves
Machine learning based identification of keystone microbes predicting assembly patterns under microbial modulation
SCB
Deterministic
Transition of assembly patterns revealed by two-state Markov model
Stochastic
Microbial pertubation
Healthy
Diarrhea
