# Supplementary material for: Varied microbial community assembly and specialization patterns driven by early life microbiome perturbation and modulation in young ruminants
Source: ISME Commun. 2024 Apr 9;4(1):ycae044. doi: 10.1093/ismeco/ycae044 (PMC11033733; doi:10.1093/ismeco/ycae044)
Supplement: Pan_et_al_Supp_figures_ISMECOMMUN-D-24-00077-final_ycae044 [file pan_et_al_supp_figures_ismecommun-d-24-00077-final_ycae044.docx]

**Fig S1.** Experimental design for the 8-week trial. The w1 (week1) to w8 (week8) stands for the start and end of this calf trial.

**Fig S2.** Characterization of longitudinal microbiome dynamic patterns using Dirichlet Multinomial Mixtures (DMM) models.

A. Characterization of dynamic microbiome patterns using the DMM model. The heatmap shows the top 10% most dominant genera based on the assignment strength. Taxa name starting with *f*_ indicate the family level of this identified microorganism.

B. The proportion of microbial communities in samples collected from different time points across each DMM cluster from w1 to w8. The size of each node is proportional to the number of samples per week. Nodes are colored according to the DMM cluster number.

C. Box plots showing the alpha diversity (Shannon and Chao1) per each DMM cluster. The centerline denotes the median, the boxes cover the 25th and 75th percentiles, and the whiskers extend to the most extreme data point, which is no more than 1.5 times the length of the box away from the box. Points outside the whiskers represent outlier samples.

**Fig S3.** Alpha diversity changes and its relations with stochasticity in both placebo-fed healthy and unhealthy calves.

A. Comparisons of alpha diversity between healthy and unhealthy groups across time using the Kruskal-Wallis test. The P-value annotated on each panel below nodes represents the significant alpha diversity between two groups (P<0.05 *). The longitudinal comparisons of alpha diversity dynamics in each group were considered significant with P<0.01 **.

B. The effects of modified stochasticity ratio (MST) on alpha diversity in healthy and unhealthy calves using mixed linear models. The small dots on each panel represent the actual data while large dots fitted into a line represent the predicted data generated by mixed linear models. The chi-square test was used to determine if alpha diversity affected MST based on the comparison of null models (only random effects) vs full models (both fixed and random effects) with *P* < 0.05 as a significance.

**Fig S4.** Microbial interactions identified from co-occurrence networks in placebo-fed healthy and diarrheic calves. Only the top 5 clusters were colored.

**Fig S5.** The shifts of microbial assembly patterns from w1 to w8 of the trial for SCB-fed healthy (blue) and diarrheic (red) calves. The shift from deterministic to stochastic-driven community assembly is predicted by the two-state Markov model.


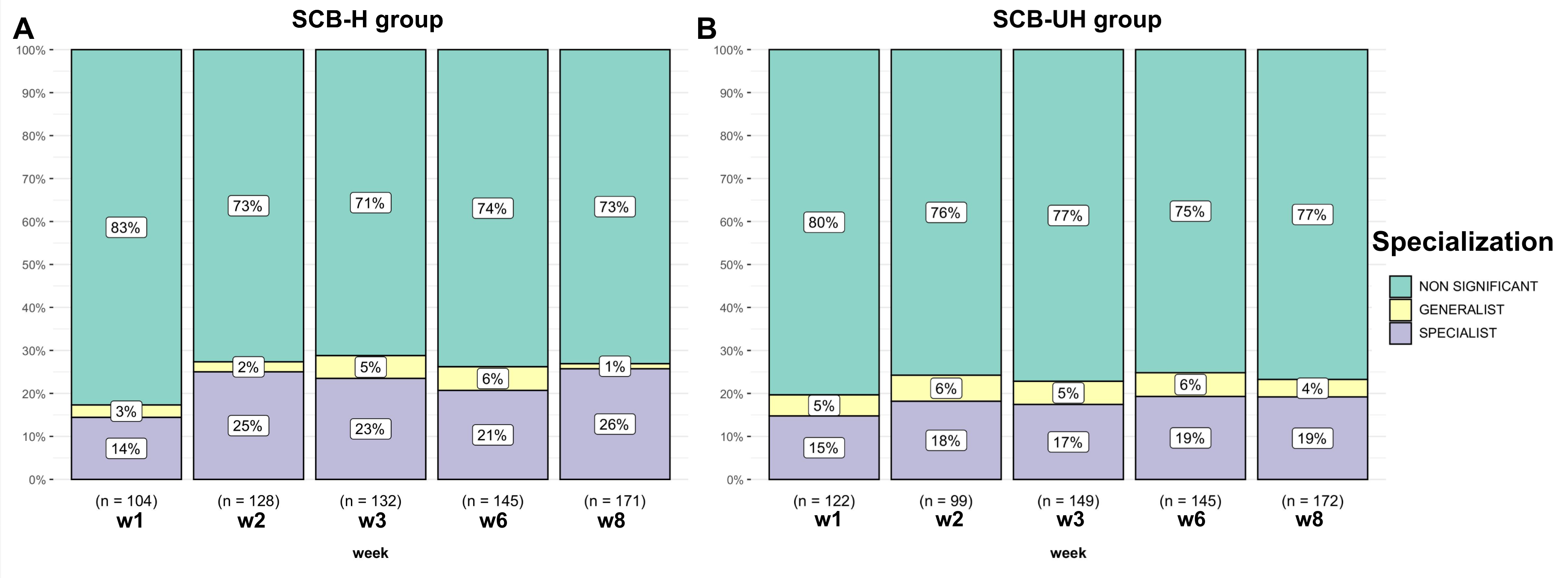


**Fig S6.** The microbial ecotypes in both SCB-fed healthy and unhealthy calves across time visualized using stack charts. Three ecotypes including generalists, specialists, and neutralists (marked as non-significant in the chart) were identified. The total number of microbial ecotypes was displayed at the bottom of each panel and the percentage of each ecotype was displayed in the chart.


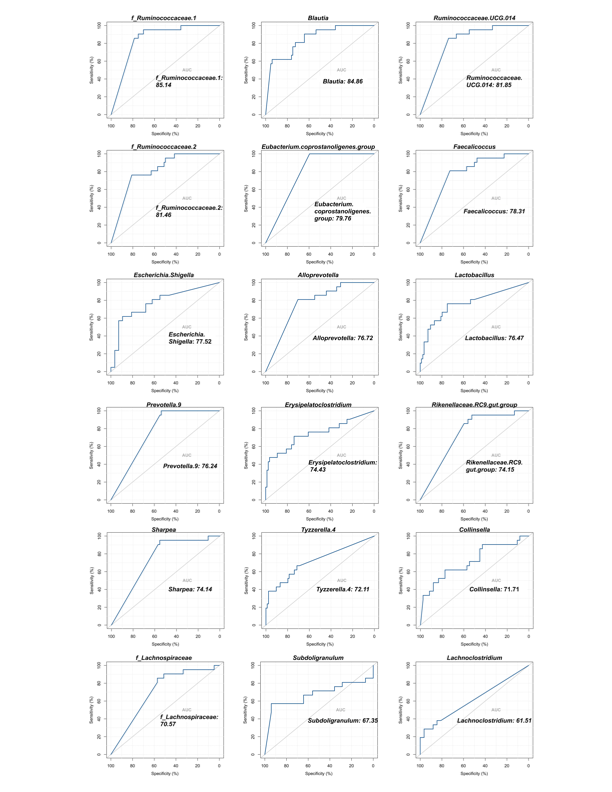


**Fig S7.** The evaluation of representative genera predicting deterministic-driven microbial assembly using the receiver operating characteristic (ROC) curve in SCB-fed healthy calves. The genera were displayed in descending order of the area under the ROC curve value.


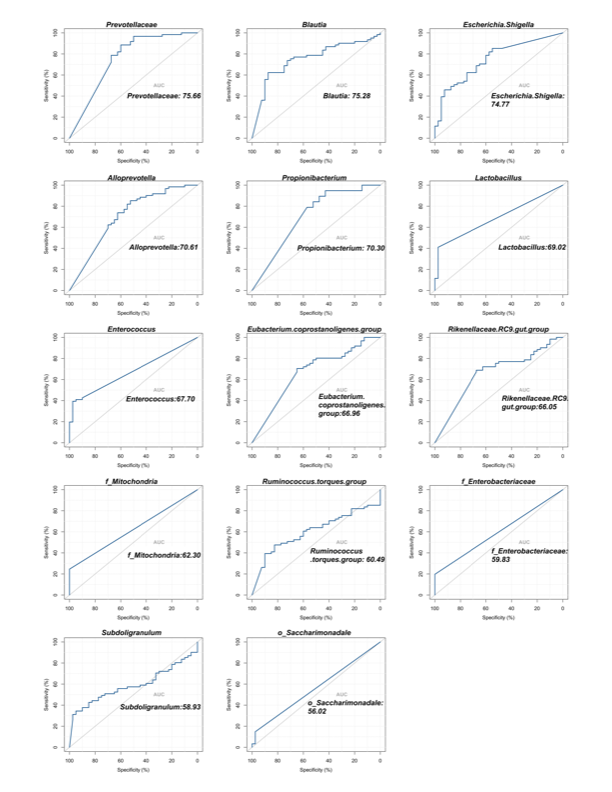


**Fig S8.** The evaluation of representative genera predicting deterministic-driven microbial assembly using the receiver operating characteristic (ROC) in SCB- fed diarrheic calves. The genera were displayed in descending order of the area under the ROC curve value.
