# Supplementary material for: Varied microbial community assembly and specialization patterns driven by early life microbiome perturbation and modulation in young ruminants
Source: ISME Commun. 2024 Apr 9;4(1):ycae044. doi: 10.1093/ismeco/ycae044 (PMC11033733; doi:10.1093/ismeco/ycae044)
Supplement: Pan_et_al_Supp_Table_ISMECOMMUN-D-24-00077-final_ycae044 [file pan_et_al_supp_table_ismecommun-d-24-00077-final_ycae044.docx]

| Week | Centroid of actual communities | Centroid of null model | *F* | *P* |
| --- | --- | --- | --- | --- |
| w1 | 0.017 | 0.014 | 0.33 | 0.57 |
| w2 | 0.011 | 0.014 | 0.19 | 0.67 |
| w3 | 0.007 | 0.002 | 4.15 | 0.04 * |
| w6 | 0.008 | 0.000 | 8.18 | <0.01* |
| w8 | 0.010 | 0.010 | 2.37 | 0.13 |

**Table S1.** The differences of centroids between the true microbial communities and null model simulations across different ages using permutational analysis of multivariate dispersions (PERMDISP) in samples collected from all calves. The significant P value represents the deterministic driven assembly (P ≤ 0.05 *).

**Table S2.** The Raup–Crick distance comparisons across ages for samples collected from all calves using pairwise PERMANOVA. The P-value was estimated by 999 permutations (P ≤ 0.01* as a significance).

| Pairwise comparison | *F* | R^2^ | *P* |
| --- | --- | --- | --- |
| w1 vs w2 | 245 | 0.6 | 0.01* |
| w1 vs w3 | 738 | 0.8 | 0.01* |
| w1 vs w6 | 725 | 0.8 | 0.01* |
| w1 vs w8 | 300 | 0.7 | 0.01* |
| w2 vs w3 | 274 | 0.6 | 0.01* |
| w2 vs w6 | 332 | 0.7 | 0.01* |
| w2 vs w8 | 277 | 0.6 | 0.01* |
| w3 vs w6 | 89 | 0.4 | 0.51 |
| w3 vs w8 | 27 | 0.2 | 1 |
| w6 vs w8 | 95 | 0.4 | 1 |

**Table S3.** The modified stochascity ratio (MST) value for healthy and diarrheic calves from w1 to w8.

| Group | Week | MST ± SD |
| --- | --- | --- |
| H | w1 | 0.33 ± 0.24 |
| H | w2 | 0.35 ± 0.28 |
| H | w3 | 0.42 ± 0.25 |
| H | w6 | 0.61 ± 0.23 |
| H | w8 | 0.48 ± 0.29 |
| UH | w1 | 0.26 ± 0.25 |
| UH | w2 | 0.37 ± 0.22 |
| UH | w3 | 0.39 + 0.25 |
| UH | w6 | 0.52 ± 0.27 |
| UH | w8 | 0.37 ± 0.23 |

**Table S4.** Topological properties in microbial co-occurrence networks from samples collected in CON-H and CON-UH groups.

| Health status | Time | Nodes | Edges | Average degree | Clustering coefficient (Transitivity) | Modularity |
| --- | --- | --- | --- | --- | --- | --- |
| H | w1 | 97 | 285 | 5.88 | 0.82 | 0.72 |
|  | w2 | 102 | 610 | 11.96 | 0.83 | 0.53 |
|  | w3 | 105 | 348 | 6.63 | 0.67 | 0.66 |
|  | w6 | 110 | 287 | 5.22 | 0.53 | 0.68 |
|  | w8 | 139 | 667 | 9.60 | 0.56 | 0.59 |
| UH | w1 | 117 | 513 | 8.77 | 0.75 | 0.50 |
|  | w2 | 102 | 418 | 8.2 | 0.80 | 0.51 |
|  | w3 | 124 | 382 | 6.16 | 0.63 | 0.61 |
|  | w6 | 121 | 349 | 5.77 | 0.64 | 0.74 |
|  | w8 | 157 | 406 | 5.17 | 0.61 | 0.74 |

| Health status | Time | Significance of average degree comparisons | Significance of clustering coefficient comparisons |
| --- | --- | --- | --- |
| H | w1 vs.w2 | <0.01 * | <0.01 * |
|  | w1 vs.w3 | 0.17 | <0.01 * |
|  | w1 vs.w6 | 0.03 * | <0.01 * |
|  | w1 vs.w8 | <0.01 * | <0.01 * |
|  | w2 vs.w3 | <0.01 * | <0.01 * |
|  | w2 vs.w6 | <0.01 * | <0.01 * |
|  | w2 vs.w8 | <0.01 * | <0.01 * |
|  | w3 vs.w6 | <0.01 * | 0.03 * |
|  | w3 vs.w8 | <0.01 * | <0.01 * |
|  | w6 vs.w8 | <0.01 * | <0.01 * |
| UH | w1 vs.w2 | 0.18 | 0.7 |
|  | w1 vs.w3 | 0.02 * | <0.01 * |
|  | w1 vs.w6 | <0.01 * | <0.01 * |
|  | w1 vs.w8 | <0.01 * | <0.01 * |
|  | w2 vs.w3 | 0.05 * | <0.01 * |
|  | w2 vs.w6 | <0.01 * | <0.01 * |
|  | w2 vs.w8 | <0.01 * | <0.01 * |
|  | w3 vs.w6 | 0.08 | 0.23 |
|  | w3 vs.w8 | <0.01 * | 0.1 |
|  | w6 vs.w8 | 0.01 * | <0.01 * |

**Table S5.** Pairwise comparisons of node distributions using non-parametric Kolmogorov-Smirnov test (P≤ 0.05 * as a significance).

**Table S6.** The influential taxa identified using the integrated value of influence (IVI) from microbial interactions in placebo-fed healthy calves (CON-H).

| Group | Week | Phyla | Genus | IVI value |
| --- | --- | --- | --- | --- |
| H | w1 | Firmicutes | *Clostridium sensu stricto 1* | 0.93 |
|  |  |  | *Lachnoclostridium* | 0.78 |
|  |  | Others | *Fusobacterium* | 0.81 |
|  |  | Proteobacteria | *Escherichia-Shigella* | 1 |
|  | w2 | Firmicutes | *Clostridium sensu stricto 1* | 0.45 |
|  |  |  | *f_Lachnospiraceae 1* | 0.35 |
|  |  |  | *Faecallibacterium* | 0.32 |
|  |  |  | *Moglbacterium* | 1 |
|  | w3 | Bacteroidetes | *Alistipes* | 0.69 |
|  |  |  | *Butyricimonas* | 0.35 |
|  |  |  | *Parabacteroides* | 1 |
|  |  | Firmicutes | *f_Ruminococcaceae 1* | 0.39 |
|  |  |  | *UBA 1819* | 0.34 |
|  | w6 | Actinobacteria | *Collinsella* | 1 |
|  |  | Firmicutes | *Butyricicoccus* | 0.38 |
|  |  |  | *f_Ruminococcaceae 1* | 0.89 |
|  |  |  | *f_Ruminococcaceae 2* | 0.61 |
|  |  |  | *Intestinibacter* | 0.49 |
|  |  |  | *Turicibacter* | 0.39 |
|  | w8 | Bacteroidetes | *Parabacteroides* | 0.60 |
|  |  |  | *Prevotella 2* | 1 |
|  |  | Firmicutes | *Anaerofilum* | 0.62 |
|  |  |  | *Dorea* | 0.78 |
|  |  |  | *Fournierella* | 0.87 |
|  |  |  | *Negativibacillus* | 0.67 |

| Group | Week | Phyla | Genus | IVI Value |
| --- | --- | --- | --- | --- |
| UH | w1 | Firmicutes | *Dorea* | 0.58 |
|  |  |  | *Faecallibacterium* | 1 |
|  |  | Proteobacteria | *Escherichia-Shigella* | 0.67 |
|  | w2 | Firmicutes | *[Ruminococcus] torques group* | 0.49 |
|  |  |  | *Erysipelatoclotridium* | 0.39 |
|  |  | Others | *Fusobacterium* | 0.59 |
|  |  | Proteobacteria | *Escherichia-Shigella* | 1 |
|  | w3 | Firmicutes | *f_Lachnospiraceae 2* | 1 |
|  |  |  | *f_Ruminococcaceae 2* | 0.77 |
|  |  |  | *Ruminiclostridium 9* | 0.43 |
|  |  | Proteobacteria | *Sutterella* | 0.62 |
|  | w6 | Actinobacteria | *f_Atopobiaceae* | 0.78 |
|  |  |  | *Truperella* | 0.54 |
|  |  | Bacteroidetes | *Alistipes* | 0.83 |
|  |  |  | *dgA-11 gut group* | 0.61 |
|  |  |  | *f_Flavobacteriaceae* | 0.52 |
|  |  | Firmicutes | *Butyricicoccus* | 0.72 |
|  |  |  | *Catabacter* | 0.54 |
|  |  |  | *Christensenellaceae R-7 group* | 0.87 |
|  |  |  | *Epulopiscium* | 0.54 |
|  |  |  | *f_Ruminococcaceae 1* | 0.51 |
|  |  |  | *f_Ruminococcaceae 2* | 0.70 |
|  |  |  | *GCA 900066225* | 1 |
|  |  |  | *Howardella* | 0.54 |
|  |  |  | *Parvimos* | 0.54 |
|  |  |  | *Ruminococcaceae UCG-005* | 0.96 |
|  |  |  | *Terrisporobacter* | 0.54 |
|  |  | Others | *c_Saccharimodia 1* | 0.54 |
|  |  |  | *Cloacibacillus* | 0.54 |
|  | w8 | Bacteroidetes | *Alistipes* | 0.37 |
|  |  |  | *Bacteroides* | 1 |
|  |  |  | *Parabacteroides* | 0.67 |
|  |  | Firmicutes | *Dorea* | 0.46 |
|  |  |  | *f_Ruminococcaceae 2* | 0.37 |
|  |  |  | *Intestinimonas* | 0.44 |
|  |  |  | *Negativibacillus* | 0.70 |
|  |  |  | *Tyzzerella* | 0.49 |

**Table S7.** The influential taxa identified using the integrated value of influence (IVI) from microbial interactions in placebo-fed diarrheic calves (CON-UH)

**Table S8.** Predicting genera for assembly patterns using Boruta method in SCB-fed healthy and diarrheic calves.

| Group | Phyla | Genus |
| --- | --- | --- |
| H | Actinobacteria | *Collinsella* |
|  | Bacteroidetes | *Alloprevotella; Prevotella 2; Prevotella 9;*  *Rikenellaceae RC9 gut group* |
|  | Firmicutes | *Lactobacillus; Clostridium sensu stricto 2;*  *Parvimonas; Blautia; Lachnoclostridium;*  *Tyzzerella 4; Lachnospiraceae;*  *Ruminococcaceae UCG 14; Subdoligranulum*  *Eubacterium coprostanoligenes group;*  *f_Ruminococcaceae 1;f_Ruminococcaceae 2*  *Erysipelatoclostridium; Faecalicoccus; Sharpea* |
|  | Proteobacteria | *Escherichia Shigella; Gallibacterium* |
|  | Verrucomicrobia | *Akkermansia* |
| UH | Actinobacteria | *Propionibacterium* |
|  | Bacteroidetes | *Alloprevotella; Prevotella 2; Prevotella 9; f_Prevotellaceae; Rikenellaceae RC9 gut group;*  *dgA 11 gut group* |
|  | Firmicutes | *Enterococcus; Lactobacillus; Anaerostipes;Blautia;*  *Sellimonas;Ruminococcus torques;Subdoligranulum; Eubacterium coprosta; f_Ruminococcaceae 1* |
|  | Patescibacteria | *c_Saccharimonadales* |
|  | Proteobacteria | *f_Mitochondria; Escherichia Shigella;*  *f_Enterobacteriaceae* |
